# Mechanisms determining the multi-diversity of carrion visiting species along a gradient of carrion body mass

**DOI:** 10.1007/s00442-024-05611-1

**Published:** 2024-09-09

**Authors:** Amelie Wierer, Christian von Hoermann, M. Eric Benbow, Christiane Büchner, Heike Feldhaar, Christian Fiderer, Oliver Mitesser, Janine Rietz, Jens Schlüter, Johannes Zeitzler, Tomáš Lackner, Claus Bässler, Marco Heurich, Jörg Müller

**Affiliations:** 1 Chair of Conservation Biology and Forest Ecology, Biocenter University of Würzburg, Glashüttenstr. 5, 96181 Rauhenebrach, Germany; 2 Department of Conservation and Research, Bavarian Forest National Park, Freyunger Str. 2, 94481 Grafenau, Germany; 3 Department of Entomology, Department of Osteopathic Medical Specialties; Ecology, Evolution and Behavior Program, AgBioResearch, Michigan State University, East Lansing, MI USA; 4 Animal Ecology I, Bayreuth Center of Ecology and Environmental Research, University of Bayreuth, 95440 Bayreuth, Germany; 5 Department of National Park Monitoring and Animal Management, Bavarian Forest National Park, Freyunger Straße 2, 94481 Grafenau, Germany; 6 Weihenstephan-Triesdorf University of Applied Sciences, Am Hofgarten 4, 85354 Freising, Germany; 7 Institute of Forestry and Wildlife Management, Inland Norway University of Applied Science, NO-2480 Koppang, Norway; 8 Fungal Ecology, Bayreuth Center of Ecology and Environmental Research, University of Bayreuth, 95440 Bayreuth, Germany

**Keywords:** Experimental carrion ecology, Bavarian Forest National Park, Necromass

## Abstract

Resource availability and habitat heterogeneity are essential drivers of biodiversity, but their individual roles often remain unclear since both factors are often correlated. Here, we tested the *more-individuals hypothesis* (MIH) and the *habitat-heterogeneity hypothesis* (HHH) for bacteria, fungi, dipterans, coleopterans, birds, and mammals on 100 experimentally exposed carcasses ranging by three orders of magnitude in body mass. At the level of each carcass we found marginal or significant support for the MIH for bacteria, fungi, and beetles in spring and significant support for fungi, dipterans, and mammals in summer. The HHH was supported only for bacteria in spring, while it was supported for all groups except mammals in summer. Overall multidiversity always increased with body mass, with a steeper increase in summer. Abundance based rarefaction-extrapolation curves for three classes of body mass showed the highest species richness for medium-sized carcasses, particular for dipterans and microbes, supporting the HHH also among carcasses. These findings complement existing necromass studies of deadwood, showing there are more niches associated with larger resource amounts and an increasing habitat heterogeneity between carcasses most pronounced for medium-sized species. Higher resource amount led to increased diversity of carrion-consuming organisms in summer, particularly due to the increasing number of niches with increasing size. Our findings underline the importance of distributed large carrion as well as medium-sized carrion in ecosystems supporting overall biodiversity of carrion-consumers. Furthermore, the different responses in spring and summer may inform strategies of carrion enrichment management schemes throughout the year.

**Supplementary Information:**

The online version contains supplementary material available at 10.1007/s00442-024-05611-1.

## Introduction

Biodiversity influences ecosystem functions such as decomposition, during which dead organic matter, the so-called necromass, is broken down while energy and nutrients are recycled (Cardinale et al. 2012). In addition, decomposition limits biomass accumulation by removing dead organisms, which also reduces the risk of disease transmission (Benbow et al. 2019; Newsome et al. 2021). Conserving the biodiversity of the organisms involved in decomposition is essential for maintaining their important role in ecosystem functioning (Carter et al. 2007; Delgado-Baquerizo et al. 2020; Newsome et al. 2021). Within the “necrobiome” (a holistic concept that summarizes necromass and its decomposer communities), most studies have thus far focused on the decomposition of plant necromass and its importance for biodiversity (Seibold et al. 2016; Benbow et al. 2019; Müller et al. 2020).

In contrast to other kinds of necromass, animal necromass (carrion) has been less studied and the ecological mechanisms of biodiversity patterns are less understood. Carrion is a prevalent form of biomass that provides an intense, localized nutrient pulse due to its fast turnover and high nutrient concentration (i.e., high content of fatty acids, nitrogen, and phosphorus; Carter et al. 2007; Barton et al. 2013; Benbow et al. 2019, Olea et al. 2019). Carrion consumers are generally divided into scavengers (taxa that feed on carrion), and saprotrophic decomposers (bacteria and fungi) that use carrion as an in situ habitat and nutrient source (Jones et al. 2015; von Hoermann et al. 2022). Carrion-associated vertebrates are either obligate (e.g., vultures) or facultative scavengers (i.e., complementing their diet by consuming carrion, e.g., most predatory mammals; Pereira et al. 2014). Few species rely predominantly on carrion as a food source and most shift between predation and scavenging (DeVault et al. 2003; Pereira et al. 2014; Jones et al. 2015). After very diverse microbes, arthropods are the most species-rich and abundant taxon that use carrion (Barton et al. 2013). Beetles utilize carrion for feeding and as a reproductive resource (Beninger & Peck 1992). Some beetle species are necrophagous and others are parasitoid or predatory species drawn to carcasses by the presence of, e.g., Diptera larvae (Jones et al. 2015; von Hoermann et al. 2018; Weithmann et al. 2021). Diptera also breed on carrion, and while the adults often utilize other food sources, the larvae feed on the carcass and accelerate early decomposition by secreting digestive enzymes that dissolve soft tissue (Prado e Castro et al. 2012; Jones et al. 2015). Due to the differing usage, the distinct groups of carrion visitors depend on carrion to different extents, but as they utilize the same resource, there should be intense competition among vertebrates, arthropods, and microbial decomposer taxa (DeVault et al. 2003; Rozen et al. 2008).

According to the broader understanding of species-area relationships (the general pattern of increasing species richness with increasing habitat area; MacArthur & Wilson 1967; Seibold et al. 2016), the simultaneous use by multiple carrion-visiting taxa is more likely if the resource area (i.e., the size of the carcass) is larger. Ecological theory suggests that species-area relationships can be explained by resource or energy availability, represented by carcass size (Hurlbert 2006; Seibold et al. 2016), and habitat heterogeneity. The *more-individuals hypothesis* (MIH) is a variant of the species energy hypothesis (Wright 1983; Clarke & Gaston 2006) and posits that energy availability from resources increases with area (i.e., carcass body mass). This leads to an increase in the abundance of organisms that utilize the resource and an increase in the abundance of organisms (e.g., Silphidae, Staphylinidae) that prey on the feeding organisms (e.g., maggots). A higher number of individuals implies a higher number of species because species richness is an increasing function of total abundance (e.g., a larger resource supports a greater number of different populations, so more species can coexist; Srivastava & Lawton 1998; Müller et al. 2014; Schuler et al. 2015; Seibold et al. 2015). According to the *habitat-heterogeneity hypothesis* (HHH), an increase in resource area leads to a higher number of habitats and ecological niches. With the prerequisite that different species use different niches, the species richness increases when heterogeneous habitats are available (e.g., within a large carcass, various decomposition stages exist simultaneously; Tews et al. 2004; Seibold et al. 2016; Müller et al. 2018; Benbow et al. 2019).

To test the *more-individuals hypothesis* and the *habitat-heterogeneity hypothesis* for the diversity of carrion visitors, we experimentally exposed 100 carcasses of ten different mammal species during spring and summer in a low mountain forest range in Central Europe with a rich scavenger community. We quantified abundance and species richness for bacteria, fungi, dipterans, beetles, birds, and mammals. For the first time for carrion necromass, we used an exhaustive multi-taxa approach to test multidiversity across bacteria, fungi, dipterans, beetles, birds, and mammals against carrion body mass.

## Materials and methods

### Study area and experimental design

**Fig. 1:**
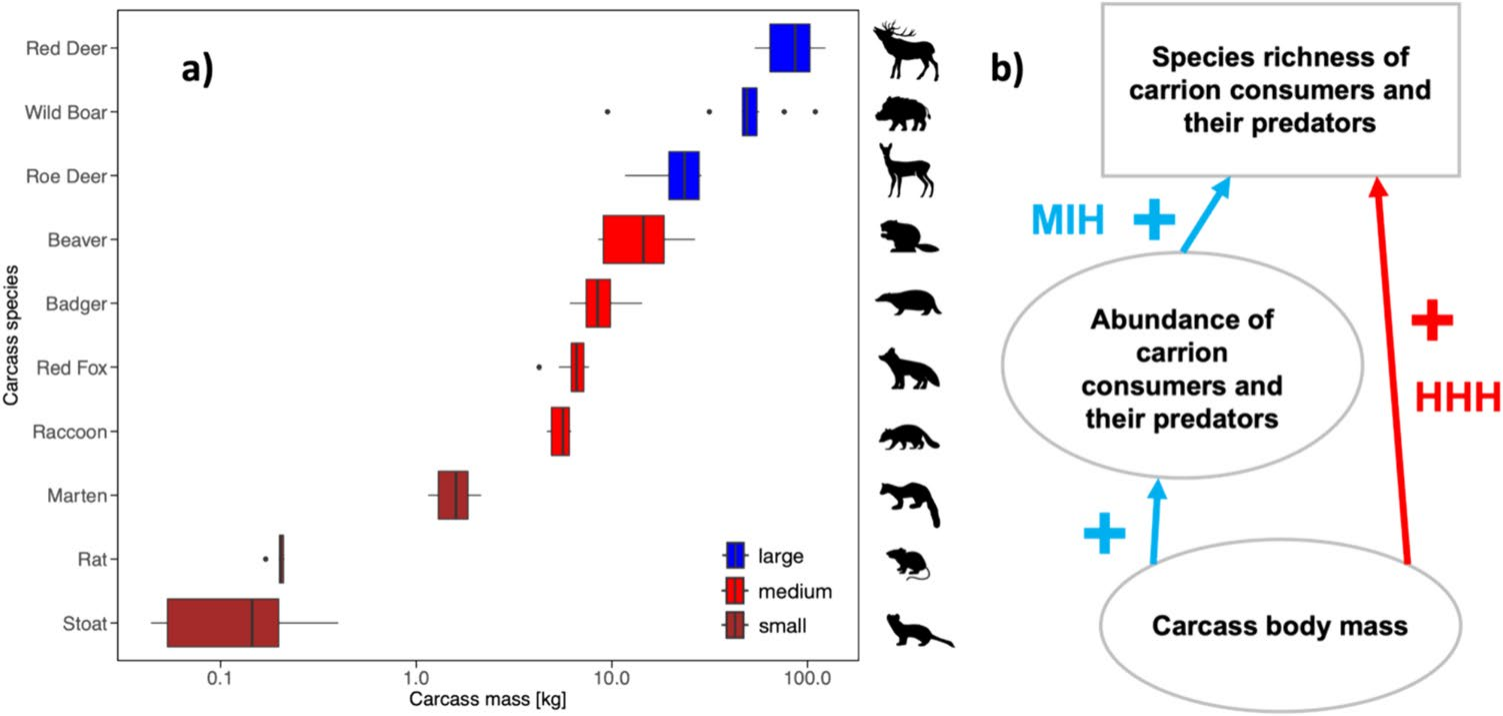
**a** Carcass body mass (in kg, logarithmic scaling, base 10) of ten different mammal species ranging from 0.044 kg (single stoat carcass) to 123.6 kg (single red deer carcass, Appendix S1: Table S1). **b** Conceptual figure for comparing the MIH and HHH. If the HHH applies, we expect a direct positive effect of carcass body mass on species richness (red arrow); if the MIH applies, we expect an indirect positive effect of carcass body mass on species richness through a positive effect on abundance (blue arrows)

Our study was conducted in semi-open mixed montane forest stands and subalpine spruce stands of the Bavarian Forest National Park (BFNP) in southeastern Germany. The BFNP is a low-range mixed mountain forest (Heurich et al. 2010), and the habitat of herbivores such as roe deer (*Capreolus capreolus*), red deer (*Cervus elaphus*), and European beaver (*Castor fiber*), carnivores such as Eurasian lynx (*Lynx lynx*) and gray wolf (*Canis lupus*), red fox (*Vulpes vulpes*), marten (*Martes martes/foina*) and omnivores such as wild boar (*Sus scrofa*) and European badger (*Meles meles*). The temperate climate is characterized by cold and humid conditions, with maritime influence from the west; mean air temperatures range between 3.9 and 8.6 °C, and the annual precipitation varies from 1400 up to 2500 mm (von Hoermann et al. 2021). In spring (April to May) and in summer (July) of 2021 (Appendix S1: Table S1) we exposed ten different mammal carrion species with body masses ranging from 0.044 kg (single stoat carcass) to 123.6 kg (single red deer carcass, Fig. 1a, Appendix S1: Table S1). The ten carrion species were exposed in a randomized order along a linear transect at each of the five exposition sites (Appendix S1: Fig. S1), with a minimum intercarcass distance of 100 m to reduce potential cross-contamination among carcasses and facilitate independence of replicates (Schoenly et al. 1991; Perez et al. 2016). A minimum distance of 80 m to trails was used to prevent disturbance by humans. The summer deployment was carried out as repeated baiting, and a new carcass of the same species was placed about 5 m next to the remains of the previous carcass from the spring deployment. The elevation of the experimental plots ranged from 734 to 1286 m a.s.l. As carrion species, we selected stoat (*Mustela erminea/nivalis*), Norway rat (*Rattus norvegicus*), marten, raccoon (*Procyon lotor*), European badger, red fox, European beaver, roe deer, wild boar, and red deer to represent a wide range of diets and body mass (Fig. 1a). All carcasses were stored frozen and defrosted for periods of 24 h up to 5 days, depending on the body mass prior to the day of exposure (day 0). The ten carcasses were exposed simultaneously on each site. All carcasses originated from random wildlife-vehicle collisions on National Park roads or wildlife control measures in a German Biosphere Reserve. Carcasses were allowed to decompose for an experimental period of 30 days, during which they were sampled to represent taxa visiting the resource during all morphologically distinguishable stages of the decomposition process (von Hoermann et al. 2018, 2022).

### Biodiversity sampling

For a detailed description of the biodiversity sampling see supplementary material (Appendix S2). In summary, we monitored carrion-visiting vertebrates using one camera trap per carcass, operating for 30 days. All images were processed and classified on species level with the software TRAPPER (Bubnicki et al. 2016). We used the number of independent events as a measure for the abundance of mammals and birds recorded by the camera (see Appendix S2). Carrion-associated insects were sampled with one pitfall trap installed adjacent to the mouth of each carcass to cover an important settlement area for carrion-inhabiting insects (Dekeirsschieter et al. 2011; von Hoermann et al. 2021, 2023). During the decomposition period, a total of four collection events of 48 h were conducted on days 4, 8, 16, and 23 after exposure to cover all decomposition stages. We separated carrion beetles from the families Dermestidae, Histeridae, Scarabaeidae, Silphidae, and Staphylinidae, and identified all specimen. Diptera were collected as their larvae. Here we identified carrion-related species using DNA metabarcoding and BINs (Barcode Index Numbers), which closely approximate species (Ratnasingham and Hebert 2013). As a substitute for abundance, we used the number of reads per BIN. The carrion-inhabiting microbiome was sampled with swabs of the carcass oral mucosa on days 0, 4, 8, 16, and 23 after exposure (von Hoermann et al. 2022, 2023). The bacterial and fungal diversity in the DNA samples was characterized with amplicon sequencing (following the procedure described in von Hoermann et al. 2023). The bacterial community composition was determined using high-throughput sequencing of the 16S rRNA gene, while fungal composition was determined using high-throughput sequencing based on the internal transcribed spacer (ITS) region. Bacterial and fungal operational taxonomic units (OTUs, as a close approximation to species) were taxonomically assigned with a naïve Bayes classifier. In subsequent analyses, the number of reads per OTU was used as a substitute for abundance, as is done in routine postmortem microbiome studies (Pechal et al. 2018).

### Data analysis

All statistical analyses were conducted in R (version 4.4.1) and RStudio (version 2024.04.2 + 764, R Core Team 2024). Statistical significance was assumed for *p*-values < 0.05. For each carcass, we pooled all separate sample units from different decay stages of the same carcass to obtain one sample per carcass. For this sample, we calculated the number of species (taxonomic species for vertebrates and beetles, BINs for dipterans, and OTUs for bacteria and fungi) and the abundance (number of events for vertebrates, number of individuals for beetles, and number of reads for bacteria, fungi, and dipterans). We further calculated the multidiversity for each carcass using the function *multidiv* in R (Allan et al. 2014). The multidiversity index is a composite metric of the diversity measures (i.e., species richness) across the six taxonomic groups of carrion-visiting organisms, for which each taxon is weighted equally (Allan et al. 2014; Delgado-Baquerizo et al. 2020). As multidiversity ranges between 0 and 1, we fitted a beta regression model using the function *betareg* in the R package *betareg* (Cribari-Neto & Zeileis 2010) to test the effects of carcass mass on multidiversity for both seasons. To test the *more-individuals* and *habitat-heterogeneity* hypotheses (Fig. 1b) for both seasons we fitted negative binomial generalized linear models (NBGLMs, Zuur et al. 2009) using the function *glm.nb* in the R package *MASS* (Venables & Ripley 2002). In all models, *abundance* and *body mass* were log-transformed when used as predictors. First, we used *number of species* as the response variable and *body mass* of the carcass as the predictor variable to test if the number of species increased with body mass (Model 1). To test for the MIH, we fitted a model with *abundance* as the response variable and *body mass* as the predictor variable (Model 2). Indirect positive effects of *body mass* on the *number of species* through positive effects on *abundance* indicate an increase in richness via an increase in resource amount. To test for the HHH, we fitted a model with *number of species* as the response variable and *abundance* and *body mass* as predictors (Model 3). Here, positive effects of *body mass* on the *number of species* while controlling for *abundance* indicate an increase in richness via an increase in niches (Seibold et al. 2016). Furthermore, we simultaneously tested the effects for both seasons using a season-specific estimate for the predictors. We also tested for potential interactions between the six carrion-visiting groups, again using NBGLMs. We fitted a model with the *abundance* of one of the six taxa as the response variable and the *abundances* of all other single taxa as the predictor variables, and similarly a model with *species richness* of the specific taxon as the response variable and the *abundances* of all other single taxa as the predictor variables. Again, *abundance* values used as predictors were log-transformed. To estimate overall species richness among carcasses in three body mass classes for each season, we calculated rarefaction-extrapolation curves with the function *iNext* in the R package *iNext* (Hsieh et al. 2016) to compare the relationship between “abundance” (events, individuals, reads) and richness across carcasses. For this analysis, we grouped the carcass species into three categories (small: stoat, rat, and marten; medium: raccoon, red fox, badger, and beaver; large: roe deer, wild boar, and red deer) according to carcass mass (Fig. 1a).

## Results

In total, we found 13 mammal species, 16 bird species, and 64 beetle species across the 100 carcasses. Metabarcoding identified 97 Diptera BINs with a relationship to carrion (based on the families), and the amplicon sequencing produced 1364 bacterial and 1649 fungal OTUs. The mean species richness of the six groups of carrion-visiting organisms varied with carcass species, and in summer the mean carrion-visiting species richness was higher than in spring for most groups (Appendix S1: Fig. S2). Specifically, the mean carrion-visiting species numbers of mammals, birds, and Coleoptera were only higher for some carcass species in summer (e.g., roe deer, Appendix S1: Fig. S2). For Diptera, bacteria, and fungi, the mean carrion-visiting species numbers were generally higher in summer with only a few exceptions (Appendix S1: Fig. S2). The carcass species with the highest mass (red deer) mostly showed more mean visiting species than the carcass species with the lowest mass (stoat) across all six groups in both seasons (Appendix S1: Fig. S2).

**Fig. 2:**
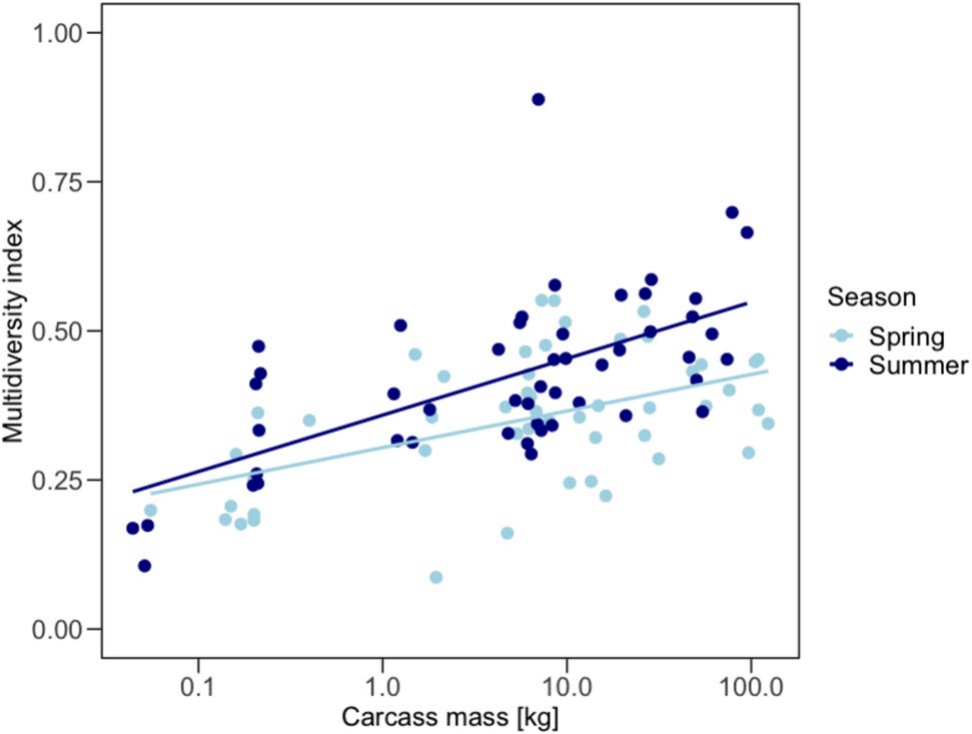
Multidiversity indices of 100 carcasses show an increase of multidiversity with carcass mass (in kg, logarithmic scaling, base 10) and a generally higher multidiversity in summer. Observed values for spring (light blue dots) and summer (dark blue dots) and predicted values (connected by the respectively colored lines) for a generalized linear abline (formula = y ~ x)

In both seasons, the overall multidiversity of all six carrion-visiting taxa combined significantly increased with carcass mass, as shown by the beta regression model (Fig. 2, Appendix S1: Table S2). In summer, multidiversity was generally higher than in spring (Fig. 2). The slope of multidiversity values was also steeper in summer than in spring, as statistically validated by a significant interaction in the model (Appendix S1: Table S2).

**Fig. 3:**
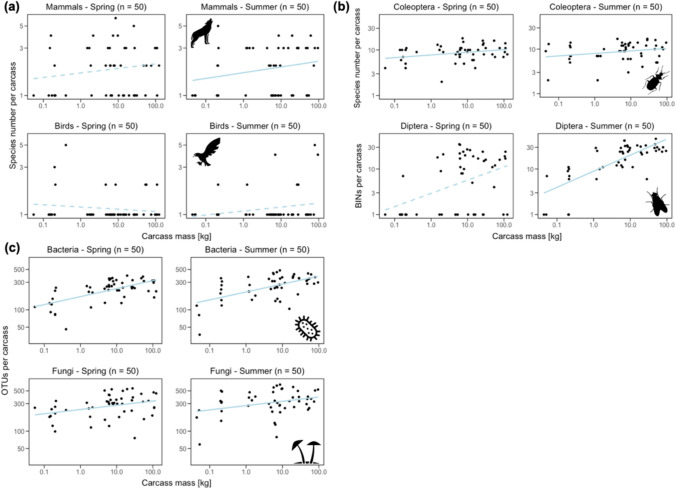
Relationship between species number/number of BINs/OTUs (species richness, logarithmic scaling, base 10) and carcass mass (in kg, logarithmic scaling, base 10) for mammals and birds (**a**), Coleoptera and Diptera (**b**), and bacteria and fungi (**c**) in spring and summer. An increase in species richness of each group with carcass mass in spring and/or summer is apparent (except birds). Observed values (black dots) and predicted values (connected by the light blue lines, dashed lines indicate an insignificant increase, see Table 1) for generalized linear ablines (formula = y ~ x). *n* represents the total number of carcasses sampled per season. Note that the y-axes for Coleoptera, Diptera, bacteria, and fungi are in different scales than for mammals and birds

The species richness of each group of carrion visitors increased with carcass mass in both seasons, except for birds in spring, where it decreased, but not significantly (Fig. 3). The negative binomial generalized linear models (NBGLMs) revealed a significant positive effect of increasing carcass mass on species richness for bacteria and fungi in spring, and mammals, Coleoptera, Diptera, bacteria and fungi in summer (Fig. 3, Table 1).

**Table 1:** Negative binomial generalized linear models (NBGLMs; Models 1, 2, and 3 in order) showing the relationship between variables studied (“Season”, “Carcass mass”, and “Abundance”) and response variables (“Species number” and “Abundance”) of the respective carrion visitor taxa

Response variable	Model	Parameter	Estimate ± SE	*z*-value	Pr( >|z|)
Species number Season:log(Carcass mass)					
Mammals	Spring:log(Carcass mass)		0.04 ± 0.04	1.07	0.2824
	Summer:log(Carcass mass)		0.09 ± 0.04	2.02	**0.0426**
Birds	Spring:log(Carcass mass)		0.03 ± 0.08	0.36	0.7188
	Summer:log(Carcass mass)		0.13 ± 0.08	1.57	0.1143
Coleoptera	Spring:log(Carcass mass)		0.04 ± 0.02	1.92	0.0543
	Summer:log(Carcass mass)		0.05 ± 0.02	2.23	**0.0253**
Diptera	Spring:log(Carcass mass)		0.04 ± 0.03	1.08	0.279
	Summer:log(Carcass mass)		0.18 ± 0.03	5.63	** < 0.001**
Bacteria	Spring:log(Carcass mass)		0.09 ± 0.02	4.42	** < 0.001**
	Summer:log(Carcass mass)		0.13 ± 0.02	6.15	** < 0.001**
Fungi	Spring:log(Carcass mass)		0.05 ± 0.02	2.07	**0.0380**
	Summer:log(Carcass mass)		0.08 ± 0.02	3.12	**0.0017**
Abundance Season:log(Carcass mass)					
Mammals	Spring:log(Carcass mass)		0.12 ± 0.08	1.51	0.1290
	Summer:log(Carcass mass)		0.25 ± 0.08	3.10	**0.0018**
Birds	Spring:log(Carcass mass)		0.05 ± 0.14	0.36	0.7191
	Summer:log(Carcass mass)		0.60 ± 0.14	4.07	**< 0.001**
Coleoptera	Spring:log(Carcass mass)		0.15 ± 0.04	3.26	**0.0011**
	Summer:log(Carcass mass)		– 0.04 ± 0.04	– 0.92	0.3534
Diptera	Spring:log(Carcass mass)		0.03 ± 0.05	0.63	0.526
	Summer:log(Carcass mass)		0.22 ± 0.04	4.50	**< 0.001**
Bacteria	Spring:log(Carcass mass)		0.04 ± 0.02	1.85	0.0637
	Summer:log(Carcass mass)		0.01 ± 0.02	0.70	0.4819
Fungi	Spring:log(Carcass mass)		0.08 ± 0.02	2.77	**0.0055**
	Summer:log(Carcass mass)		0.11 ± 0.03	3.66	**< 0.001**
Species richness log(Abundance+1) + Season:log(Carcass mass)					
Mammals	log(Abundance+1)		0.43 ± 0.06	6.97	**< 0.001**
	Spring:log(Carcass mass)		0.01 ± 0.04	0.06	0.947
	Summer:log(Carcass mass)		– 0.01 ± 0.04	– 0.27	0.782
Birds	log(Abundance+1)		0.74 ± 0.09	8.01	**< 0.001**
	Spring:log(Carcass mass)		0.01 ± 0.07	0.03	0.9696
	Summer:log(Carcass mass)		– 0.18 ± 0.08	– 2.17	**0.0295**
Coleoptera	log(Abundance)		0.27 ± 0.03	7.14	**< 0.001**
	Spring:log(Carcass mass)		– 0.01 ± 0.02	– 0.49	0.6215
	Summer:log(Carcass mass)		0.05 ± 0.02	2.78	**0.0053**
Diptera	log(Abundance)		0.21 ± 0.06	3.45	**< 0.001**
	Spring:log(Carcass mass)		0.02 ± 0.03	0.57	0.5660
	Summer:log(Carcass mass)		0.12 ± 0.03	3.54	**< 0.001**
Bacteria	log(Abundance)		0.32 ± 0.07	4.29	**< 0.001**
	Spring:log(Carcass mass)		0.07 ± 0.02	3.59	**< 0.001**
	Summer:log(Carcass mass)		0.13 ± 0.02	6.28	**< 0.001**
Fungi	log(Abundance)		0.28 ± 0.07	3.85	**< 0.001**
	Spring:log(Carcass mass)		0.02 ± 0.02	1.17	0.2394
	Summer:log(Carcass mass)		0.05 ± 0.02	2.02	**0.0426**

The estimate of the parameters, the standard error of the parameters (SE), the z-value, and the p-value (Pr( >|z|)) are shown. Significant *p*-values (< 0.05) are indicated in boldface

The effect was marginally significant for Coleoptera in spring (Fig. 3, Table 1). The models also showed a significant increase in abundance with carcass mass for Coleoptera and fungi in spring, and mammals, birds, Diptera, and fungi in summer, in line with the MIH (Fig. 3, Table 1). The increase was marginally significant for bacteria in spring (Table 1). Final NBGLMs for the number of species modeled by abundance and body mass showed positive effects of body mass after controlling for abundance, in line with the HHH (Fig. 3, Table 1) for bacteria in spring, and birds, Coleoptera, Diptera, bacteria, and fungi in summer (Table 1). The models that tested for potential negative effects between the six carrion-visiting groups through competition revealed no significant results with one exception. The number of Diptera species decreased with increasing Coleoptera abundance (Appendix S1: Table S3). Most of the observed effects between the groups were positive (Appendix S1: Table S3), supporting more facilitation than competition between groups.

**Fig. 4:**
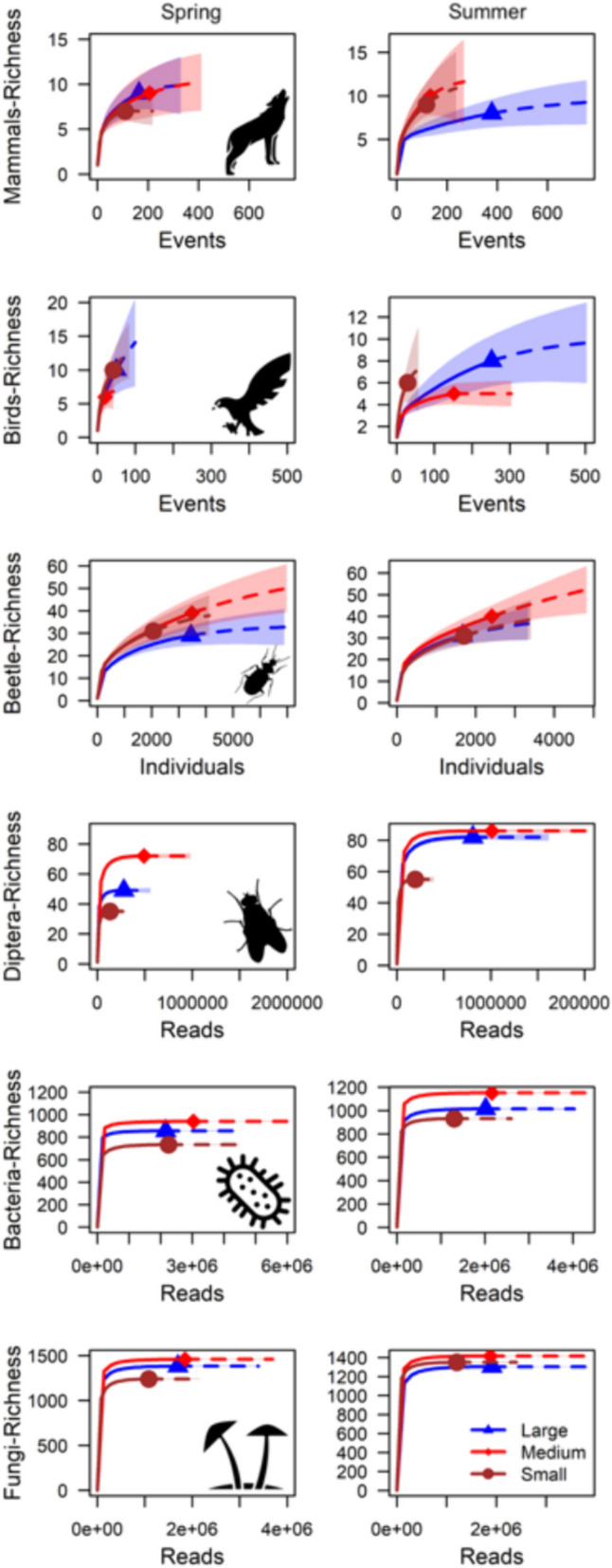
Species rarefaction-extrapolation curves for three carcass categories (small: dark red, medium: light red, and large: dark blue) show the highest overall species richness standardized by “abundance” in medium carcasses for most taxa in both seasons; followed by large and small carcasses. For birds, this order is reversed in both seasons, and for beetles in spring and mammals and fungi in summer, small carcasses are species richer than large

After standardization of species richness values for sampling effort in terms of events, individuals or reads among carcasses of the three body size categories, most taxa showed the highest richness in medium carcasses, followed by large and then small carcasses in both seasons. In some instances, namely beetles in spring and mammals and fungi in summer, small carcasses showed a higher richness than large ones. Birds provided an exception, as small carcasses showed a higher richness than large and medium carcasses in both seasons (Fig. 4).

## Discussion

Our large-scale, multi-species carrion decomposition experiment with carcass body mass representing three orders of magnitude difference provided some support for the *more-individuals hypothesis*, with more species at larger carcasses for half of the taxonomic groups in spring and summer. Furthermore, controlling the models for abundances revealed support for the *habitat-heterogeneity hypothesis* for most taxa particularly in summer (Table 2).

**Table 2:** Mechanisms explaining an increase in species richness with carcass mass for each carrion-visiting taxon

	Spring		Summer	
Taxon	MIH	HHH	MIH	HHH
Mammals	/	/	✔	/
Birds	/	/	/	✔
Coleoptera	(✔)	/	/	✔
Diptera	/	/	✔	✔
Bacteria	✔	✔	/	✔
Fungi	(✔)	/	✔	✔

MIH = *more-individuals hypothesis*, HHH = *habitat-heterogeneity hypothesis*. =  ✔ hypothesis applies with significance, (✔) = hypothesis applies with marginal significance, / = no significant support for hypothesis

The result of the highest overall species richness in medium-sized carcass species is another support for the HHH. Here it seems that the different medium-sized carrion species form a more heterogeneous landscape of carrion even than the large ones for most taxa, particularly for microbes and Diptera.

We included all parts of the necrobiome, encompassing vertebrate scavengers, insects, and microbes, for a complete understanding of the complex carrion-associated taxonomic diversity (von Hoermann et al. 2023). Previous studies have shown that carcass exposure scheme affected vertebrate and insect diversity differently, with a lower diversity of vertebrates, but a higher diversity of insects in temporally aggregated carcasses (Wilmers et al. 2003; Cortés-Avizanda et al. 2012; von Hoermann et al. 2021). In accordance with these findings, our six taxonomical groups were differently affected by season and different mechanisms behind the effect of carcass body mass on diversity.

The mean species richness of all observed carrion-associated taxa varied among carcass species, and the largest carcass species (red deer) was visited by more species than the smallest carcass species (stoat) in both seasons, as expected (Appendix S1: Fig. S2). In some cases, even smaller carcass species attracted more visiting species than larger ones; therefore, small carcasses also supported diversity. Tissue type preferences by some necrophilous species may explain this finding. For instance, a higher body fat to muscle tissue ratio, as found in beaver carcasses, can increase the diversity of carrion-associated beetles by attracting dermestid beetle species, which rely on fat tissue decomposition products for pheromone production (von Hoermann 2023, unpublished results). The importance of small- and medium-sized carcasses is further supported by the rarefaction-extrapolation curves (Fig. 4) that underline the importance of medium-sized species for landscape wide species richness, except for birds. Here it seems they offer enough resource quantity but also heterogeneity. As the “abundances” in these groups showed very similar total numbers in medium and large carrion species (Fig. 4), the higher richness in medium-sized carrion might be due to relaxed competition as shown for fungi in fine woody debris compared to coarse woody debris (Heilmann and Christensen 2004) or by higher habitat heterogeneity among medium-sized carrion, as small ones dry out fast and large ones are strongly dominated by their own mass, while medium carrion might be more variably affected by local habitat conditions (Müller et al. 2024).

Without consideration of carcass species as separate categories, we found a higher local number of species at carcasses with a higher mass in both seasons for all taxa except birds in spring. This result is in accordance with VanLaerhoven et al. (2015), Benbow et al. (2015), Turner et al. (2017), Naves-Alegre et al. (2021), and von Hoermann et al. (2021), who described an increase in carrion-associated species diversity with an increase in carrion mass. However, in our study, small carcasses also exhibited a high number of species for mammals and birds in spring. The consumption of small carcasses by multiple mesopredators might explain this, whereas large carcasses are mainly consumed by dominant scavenger species (Naves-Alegre et al. 2021). Additionally, a smaller surface area relative to volume in large resources can limit initial accessibility for some decomposers (Benbow et al. 2019). The multidiversity among all taxa increased significantly with carcass mass in both seasons, suggesting that carcasses with a high mass are especially effective in maintaining a higher broad ecosystem diversity. This is also shown by little difference between the residuals of all multidiversity values grouped by the different carrion trophic levels (carnivore, herbivore, and omnivore, Appendix S1: Fig. S3).

Our finding of a generally higher mean species richness in summer for Diptera, bacteria, and fungi (Appendix S1: Fig. S2) follows the hypothesis of higher energy availability in terms of temperature in summer (Mellard et al. 2019). The environmental conditions impacted the use of carrion resources, and summer conditions especially impacted microbes, as their metabolism activity is temperature-dependent (Carter et al. 2008; Matuszewski et al. 2011; Barton et al. 2013; Turner et al. 2017). The phenomenon of a higher diversity of microbes and insects just by warmer micro-, topo-, or macro-climate has been shown for other necromass resources, e.g., deadwood or dung (Müller et al. 2015; Milotić et al. 2019; Seibold et al. 2021; Lettenmaier et al. 2022). On the contrary, mammals and birds are likely more dependent on scavenging carrion resources in early spring (with a generally lower availability of other resources, Mellard et al. 2019), and exhibit a higher species number during this time of year (Turner et al. 2017). In summer, vertebrate facultative scavengers probably turn to other available resources than carrion, e.g., living insects or rodents with intact muscle tissue, which might explain partial decreases in species numbers in summer. However, a significantly steeper slope of multidiversity in summer suggests that despite lower species numbers for some groups, overall multidiversity rises more steeply with carrion mass than in spring.

Our models testing for both mechanisms behind an increase in the number of species with increasing carrion body mass supported both the MIH and the HHH. Interestingly, support was not randomly distributed across the six groups and the two seasons. The MIH applied for Coleoptera, bacteria*,* and fungi in spring, and mammals, Diptera, and fungi in summer, where carcass mass had a significant positive effect on both species richness and abundance. In contrast, the HHH applied only for bacteria in spring, while in summer it applied to all groups except mammals. In cases where both the MIH and the HHH were supported (bacteria in spring, Diptera and fungi in summer) an increase of carcass heterogeneity with carcass mass was likely more important than the individual effect of increased carcass amount (Seibold et al. 2016). Within a large carcass, more heterogeneous microhabitats are available to different species. For example, various decomposition stages and body-habitat-specific conditions exist simultaneously (a mosaic of decomposition stages, Braig and Perotti 2009), leading to high temperature and water content variability within one carcass. According to our results, this seems to happen more under warm summer conditions (mean air temperatures of 15.1 °C). Here, body-part-specific pathways—from rapid microbial decomposition to mummification under direct sun (used for instance by dermestid beetle larvae) to mechanical removal of body parts by scavengers such as wolf, lynx, or fox—may support higher niche diversity for larger carcasses. It is well known for several of our examined groups that niches during the insect community succession of a carcass allow the coexistence of species with similar behavior. Diptera, for example, can only be numerically dominant on carrion because the various species partition resources, which is more effective if different niches are occupied (Denno and Cothran 1976; Jones et al. 2015). This seems to explain our findings of a higher relevance of the HHH for the carrion-visiting groups that are closely bound to the decomposition process—Coleoptera, Diptera, bacteria, and fungi (Prado e Castro et al. 2012; Jones et al. 2015). The latter two microbial taxa are directly associated with carrion, acting to begin the decomposition process immediately after death (Pechal et al. 2013). Coleoptera and Diptera species are even specialized in specific phases of carcass degradation, depending on which part of the resource they consume (e.g., active and advanced decay stages with large Diptera larvae masses are preferred feeding sites for predatory Coleoptera; Lee Goff 2009, Prado e Castro et al. 2012, Jones et al. 2015, von Hoermann et al. 2021). Consequently, each of these groups benefited from the availability of a higher number of niches (Holt 2009). On the contrary, groups that rely less on carrion, such as facultatively scavenging mammals and birds, did not show a particular impact by the HHH, except for birds in summer.

In this study, resource size and heterogeneity were—as in most cases—positively correlated, which made it difficult to disentangle their respective roles in driving diversity on carrion. To understand which mechanism is more important—the MIH or the HHH—future studies should aim to minimize the correlation of resource quantity and habitat heterogeneity (e.g., Báldi 2008) by exposing different carrion species on some sites and the same carrion species on other sites as used in deadwood studies (Seibold et al. 2016), for example, to provide different levels of heterogeneity between experimental plots. Furthermore, we recommend to measure niche heterogeneity at experimentally exposed carcasses, e.g., by measurements of moisture and temperature at different parts of the carcass to distinguish between more heterogenous and homogeneous conditions within a decaying body. A very simple way might be an estimation of the different decay stages visually (Müller et al. 2024).

Furthermore, the findings from our study could be expanded in natural or anthropogenic mass mortality contexts with even greater carrion quantity, which provide an opportunity to explore the MIH and HHH further. Based on findings from deadwood created as a resource pulse by windstorms (Thorn et al. 2014), we would expect more homogenous conditions as the resource is very similar and follows simultaneous processes in space which should support the MIH, than in random mortalities with more support for the HHH. We suggest experiments mimicking both conditions in replicated landscapes.

Our findings are largely consistent with previous studies in plant necromass ecology, where evidence for both hypotheses has been reported. In a large meta-analysis, Kriegel et al. (2023) found support for the species-energy hypothesis as a mechanism for a higher saproxylic beetle diversity and specified that the relative importance of ambient temperature increases with increasing trophic level, with opposite effects for substrate energy. Seibold et al. (2016) showed an increasing saproxylic beetle diversity with an increasing amount of deadwood in sunny forests (following the MIH), whereas in shady forests, the prevailing factor was deadwood diversity (following the HHH). A more detailed study on the scale of single deadwood objects complemented these findings, demonstrating a higher saproxylic beetle diversity in logs under sunny conditions, due to a higher microclimatic heterogeneity within deadwood logs (Lettenmaier et al. 2022). This seems comparable with the findings of the two seasons in our study focusing on animal necromass. The warmer and more extreme conditions in summer (mean air temperatures of 15.1 °C), similar to deadwood in forest gaps, may create more heterogeneous habitats within carcasses than in carcasses exposed to cooler conditions in spring (mean air temperatures of 8.0 °C), similar to deadwood under closed canopy.

Of course, the communities of microbes and insects in deadwood do not always follow the same drivers, as shown by von Hoermann et al. (2023). Nevertheless, the analogies for the relevance of the MIH and the HHH in deadwood under different temperature conditions underscore the value of not limiting necromass studies to one resource type. Rather, despite very different process speeds from weeks to decades, different resources should be examined comparatively to identify general patterns and mechanisms driving consumer biodiversity (Benbow et al. 2019). More hypotheses can certainly be generated from one type of necromass and tested for others. Collectively, these studies and our results emphasize that when carrion exposition is considered to preserve the diversity of carrion-visiting taxa, complete decomposition processes are important (von Hoermann et al. 2021).

## Conclusion

Our large-scale wildlife carrion experiment demonstrates that season and body size variation among carrion species significantly affected the necrobiome diversity of carrion, with increasing species richness at a local carcass driven by resource size in accordance with the* more-individuals hypothesis* and the *habitat-heterogeneity hypothesis*. As an implication of the increase of local biodiversity with larger carcasses, but also high species richness among carcasses of medium-sized species, we suggest a strategy of mixed types of carrion distributed in the landscape, including smaller and medium-sized but also larger-bodied species such as whole red deer carcasses to provide the full niche space of a large carcass to nature, similar as in large fallen trees remaining in forests.

## Supplementary Information

Below is the link to the electronic supplementary material.

Supplementary file1 (DOCX 790 KB)

## Data Availability

Annotated R code, including the data needed to reproduce the statistical analyses and figures, is publicly available from figshare (DOI:10.6084/m9.figshare.24580810). During review, code and data are available at: https://figshare.com/s/a55135aff4fafe259aa7.
