# Supplementary material for: Mechanisms determining the multi-diversity of carrion visiting species along a gradient of carrion body mass
Source: Oecologia. 2024 Sep 9;206(1-2):115–26. doi: 10.1007/s00442-024-05611-1 (PMC11489210; doi:10.1007/s00442-024-05611-1)
Supplement: Supplementary file 1 — Supplementary file1 (DOCX 790 KB) [file 442_2024_5611_MOESM1_ESM.docx]

**Supplementary Material**

**Mechanisms determining the multi-diversity of carrion visiting species along a gradient of carrion body mass**

Amelie Wierer^1^ [
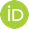
](https://orcid.org/0009-0001-1006-1371), Christian von Hoermann^1,2^ [
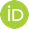
](https://orcid.org/0000-0001-6487-1540), M. Eric Benbow^4^ [
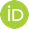
](https://orcid.org/0000-0003-2630-0282), Christiane Büchner^5^, Heike Feldhaar^5^ [
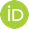
](https://orcid.org/0000-0001-6797-5126), Christian Fiderer^2,3^, Oliver Mitesser^1^ [
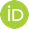
](https://orcid.org/0000-0002-3607-877X), Janine Rietz^2,3^ [
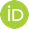
](https://orcid.org/0000-0003-1057-2304), Jens Schlüter^2^, Johannes Zeitzler^6^, Tomáš Lackner^2^ [
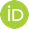
](https://orcid.org/0000-0002-0108-5785), Claus Bässler^2,5^ [
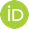
](https://orcid.org/0000-0001-8177-8997), Marco Heurich^2,3,7**^ [
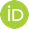
](https://orcid.org/0000-0003-0051-2930), Jörg Müller^1,2^*^,**^ [
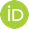
](https://orcid.org/0000-0002-1409-1586)

^1^ Field Station Fabrikschleichach, Department of Animal Ecology and Tropical Biology, Biocenter University of Würzburg, Glashüttenstr. 5, 96181 Rauhenebrach, Germany

^2^ Bavarian Forest National Park, Department of Conservation and Research, Freyunger Str. 2, 94481, Grafenau, Germany

^3^ Chair of Wildlife Ecology and Management, Albert Ludwigs University Freiburg, Tennenbacher Straβe 4, 79106, Freiburg, Germany

^4^ Department of Entomology; Department of Osteopathic Medical Specialties; Ecology, Evolution and Behavior Program; AgBioResearch; Michigan State University, East Lansing, MI, USA

^5^ Animal Ecology I, Bayreuth Center of Ecology and Environmental Research, University of Bayreuth, 95440 Bayreuth, Germany

^6^ Weihenstephan-Triesdorf University of Applied Sciences, Am Hofgarten 4, 85354 Freising

^7^ Institute of Forestry and Wildlife Management, Inland Norway University of Applied Science, NO-2480 Koppang, Norway

* Corresponding author, joerg.mueller@npv-bw.bayern.de

** Shared last authors

**Appendix S1**

**
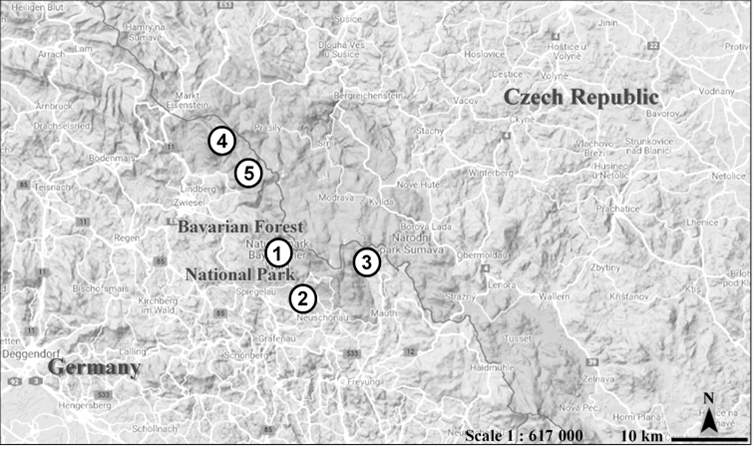
**

**Figure S1.** Map of the Bavarian Forest National Park (BFNP) with five carcass exposure sites located in semi-open mixed montane forest stands, where one set of ten carcasses per site was exposed in spring and in summer of 2021. The same five sites were used for spring and summer. Source: Prof. Dr. Jörg Müller


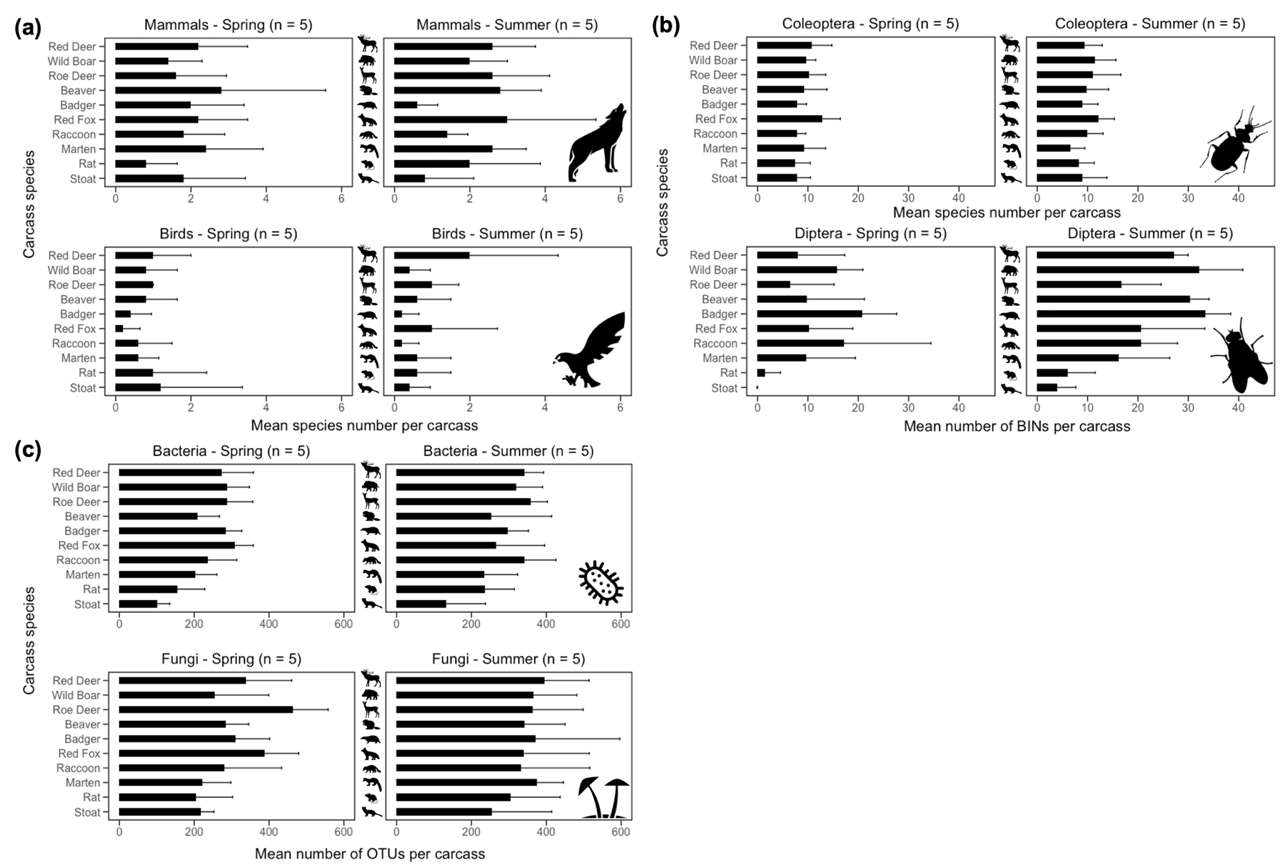


**Figure S2.** Mean species richness (with standard deviation) of mammals and birds (a), Coleoptera and Diptera (b), and bacteria and fungi (c) on each of the ten respective carcass species (sorted by decreasing mass) in spring and summer. n represents the number of carcasses of each carcass species sampled per season. Note that the x-axes for Coleoptera, Diptera, bacteria, and fungi are in different scales than for mammals and birds.


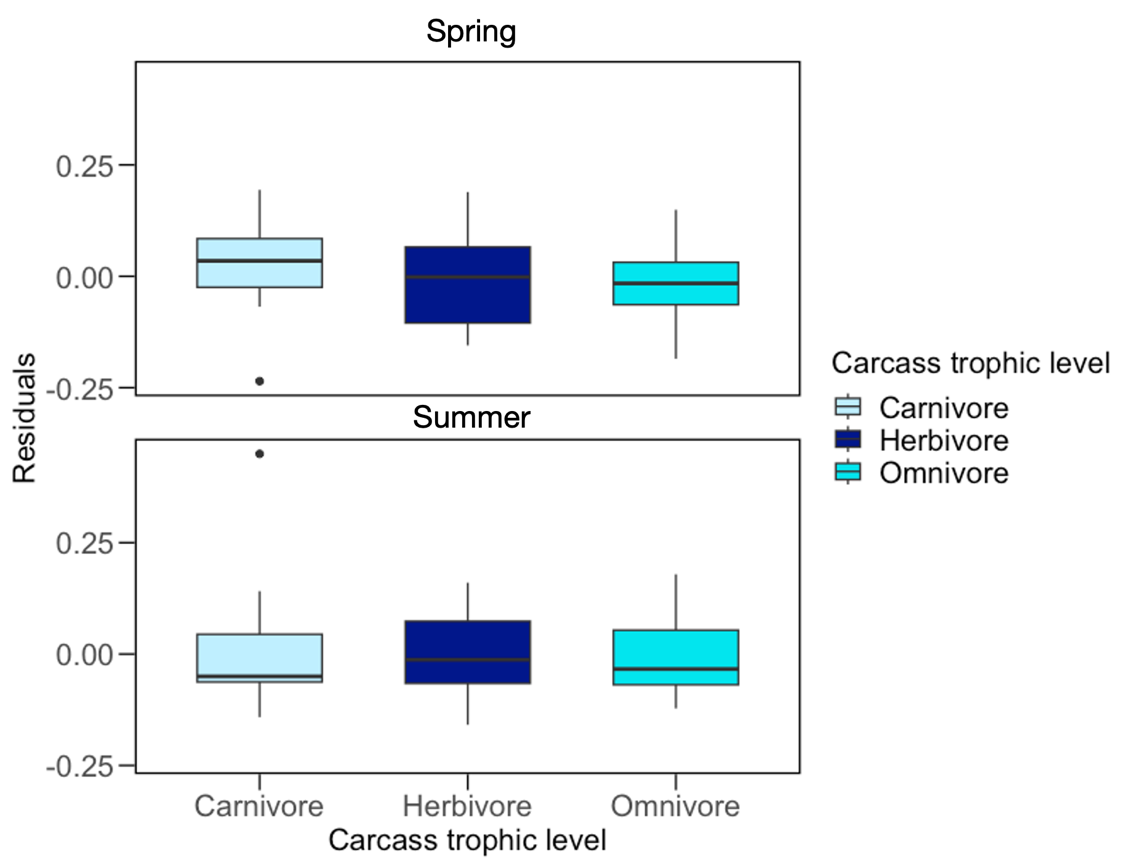


**Figure S3.** Residuals of multidiversity values from the two generalized linear ablines for the two seasons spring and summer displayed in Figure 3, grouped by carrion species trophic level (carnivore, herbivore, and omnivore). Residuals are very similar across both seasons and all trophic levels, except for one outlier for carnivores in spring, and one outlier for carnivores in summer.

**Table S1.** Carcass-ID, carcass species, carcass mass in kg, season, season block, latitude, longitude, species number and abundance of each carrion-visiting taxon, Diptera volume in ml, multidiversity, and exposition date for each of the 100 exposed carcasses.

|  | Carcass-ID | Carcass species | Carcass mass [kg] | Season | Season block | Latitude | Longitude | Birds species num-ber | Birds events | Mam-mals species num-ber | Mam-mals events |
| --- | --- | --- | --- | --- | --- | --- | --- | --- | --- | --- | --- |
| 1 | F_01_BB | European_Beaver | 8.55 | Spring | F_01 | 48.947578 | 13.406692 | 2 | 7 | 6 | 24 |
| 2 | F_01_DS | Badger | 6.1 | Spring | F_01 | 48.947463 | 13.404576 | 0 | 0 | 2 | 4 |
| 3 | F_01_FX | Red_Fox | 7.65 | Spring | F_01 | 48.94901 | 13.405459 | 0 | 0 | 2 | 6 |
| 4 | F_01_HR | Stoat | 0.4 | Spring | F_01 | 48.941375 | 13.405682 | 5 | 12 | 1 | 6 |
| 5 | F_01_MD | Marten | 2.15 | Spring | F_01 | 48.947787 | 13.405099 | 1 | 6 | 4 | 13 |
| 6 | F_01_RE | Roe_Deer | 28.1 | Spring | F_01 | 48.946788 | 13.404282 | 1 | 2 | 1 | 8 |
| 7 | F_01_RH | Red_Deer | 109.0 | Spring | F_01 | 48.942222 | 13.404772 | 2 | 9 | 3 | 50 |
| 8 | F_01_RT | Rat | 0.2 | Spring | F_01 | 48.940816 | 13.406278 | 0 | 0 | 2 | 6 |
| 9 | F_01_WB | Raccoon | 5.95 | Spring | F_01 | 48.948883 | 13.406175 | 1 | 1 | 3 | 18 |
| 10 | F_01_WS | Wild_Boar | 31.5 | Spring | F_01 | 48.946371 | 13.405046 | 0 | 0 | 1 | 9 |
| 11 | F_02_BB | European_Beaver | 26.15 | Spring | F_02 | 48.913049 | 13.444544 | 1 | 2 | 5 | 89 |
| 12 | F_02_DS | Badger | 9.8 | Spring | F_02 | 48.91484 | 13.444535 | 1 | 4 | 4 | 6 |
| 13 | F_02_FX | Red_Fox | 7.3 | Spring | F_02 | 48.915675 | 13.440239 | 1 | 1 | 4 | 14 |
| 14 | F_02_HR | Stoat | 0.055 | Spring | F_02 | 48.916027 | 13.446852 | 1 | 1 | 1 | 1 |
| 15 | F_02_MD | Marten | 1.5 | Spring | F_02 | 48.913072 | 13.445168 | 1 | 13 | 3 | 7 |
| 16 | F_02_RE | Roe_Deer | 27.5 | Spring | F_02 | 48.913619 | 13.439905 | 1 | 7 | 3 | 32 |
| 17 | F_02_RH | Red_Deer | 105.1 | Spring | F_02 | 48.912579 | 13.442123 | 0 | 0 | 3 | 17 |
| 18 | F_02_RT | Rat | 0.2 | Spring | F_02 | 48.914571 | 13.443204 | 3 | 6 | 1 | 49 |
| 19 | F_02_WB | Raccoon | 6.2 | Spring | F_02 | 48.917659 | 13.439884 | 0 | 0 | 2 | 8 |
| 20 | F_02_WS | Wild_Boar | 109.6 | Spring | F_02 | 48.916411 | 13.442673 | 0 | 0 | 0 | 0 |
| 21 | F_03_BB | European_Beaver | 13.55 | Spring | F_03 | 48.96148 | 13.559041 | 1 | 1 | 0 | 0 |
| 22 | F_03_DS | Badger | 14.3 | Spring | F_03 | 48.962026 | 13.5539 | 0 | 0 | 0 | 0 |
| 23 | F_03_FX | Red_Fox | 6.35 | Spring | F_03 | 48.963273 | 13.554227 | 0 | 0 | 1 | 7 |
| 24 | F_03_HR | Stoat | 0.16 | Spring | F_03 | 48.96307 | 13.555846 | 0 | 0 | 4 | 5 |
| 25 | F_03_MD | Marten | 1.85 | Spring | F_03 | 48.962263 | 13.560528 | 1 | 0 | 2 | 2 |
| 26 | F_03_RE | Roe_Deer | 19.5 | Spring | F_03 | 48.961411 | 13.557377 | 1 | 1 | 1 | 1 |
| 27 | F_03_RH | Red_Deer | 53.6 | Spring | F_03 | 48.96307 | 13.555846 | 2 | 6 | 0 | 0 |
| 28 | F_03_RT | Rat | 0.2 | Spring | F_03 | 48.964107 | 13.561625 | 0 | 0 | 0 | 0 |
| 29 | F_03_WB | Raccoon | 6.2 | Spring | F_03 | 48.962832 | 13.557094 | 0 | 0 | 2 | 3 |
| 30 | F_03_WS | Wild_Boar | 48.0 | Spring | F_03 | 48.963307 | 13.560377 | 1 | 8 | 2 | 2 |
| 31 | F_04_BB | European_Beaver | 16.25 | Spring | F_04 | 49.101181 | 13.297733 | 0 | 0 | 3 | 4 |
| 32 | F_04_DS | Badger | 8.0 | Spring | F_04 | 49.103043 | 13.298916 | 1 | 2 | 2 | 5 |
| 33 | F_04_FX | Red_Fox | 5.35 | Spring | F_04 | 49.100466 | 13.298425 | 0 | 0 | 3 | 7 |
| 34 | F_04_HR | Stoat | 0.14 | Spring | F_04 | 49.104047 | 13.295382 | 0 | 0 | 0 | 0 |
| 35 | F_04_MD | Marten | 1.95 | Spring | F_04 | 49.101478 | 13.296631 | 0 | 0 | 0 | 0 |
| 36 | F_04_RE | Roe_Deer | 14.8 | Spring | F_04 | 49.103798 | 13.296691 | 1 | 1 | 3 | 6 |
| 37 | F_04_RH | Red_Deer | 96.8 | Spring | F_04 | 49.103952 | 13.296187 | 0 | 0 | 3 | 4 |
| 38 | F_04_RT | Rat | 0.17 | Spring | F_04 | 49.100931 | 13.298964 | 0 | 0 | 0 | 0 |
| 39 | F_04_WB | Raccoon | 4.75 | Spring | F_04 | 49.103397 | 13.29813 | 0 | 0 | 0 | 0 |
| 40 | F_04_WS | Wild_Boar | 56.8 | Spring | F_04 | 49.101734 | 13.295547 | 2 | 1 | 2 | 27 |
| 41 | F_05_BB | European_Beaver | 10.35 | Spring | F_05 | 49.051653 | 13.366608 | 0 | 0 | 0 | 0 |
| 42 | F_05_DS | Badger | 11.7 | Spring | F_05 | 49.050845 | 13.365201 | 0 | 0 | 2 | 3 |
| 43 | F_05_FX | Red_Fox | 6.8 | Spring | F_05 | 49.052039 | 13.359878 | 0 | 0 | 1 | 2 |
| 44 | F_05_HR | Stoat | 0.15 | Spring | F_05 | 49.051576 | 13.36083 | 0 | 0 | 3 | 10 |
| 45 | F_05_MD | Marten | 1.7 | Spring | F_05 | 49.053314 | 13.355923 | 0 | 0 | 3 | 7 |
| 46 | F_05_RE | Roe_Deer | 26.45 | Spring | F_05 | 49.05303 | 13.356966 | 1 | 1 | 0 | 0 |
| 47 | F_05_RH | Red_Deer | 123.6 | Spring | F_05 | 49.052324 | 13.368634 | 1 | 1 | 2 | 6 |
| 48 | F_05_RT | Rat | 0.21 | Spring | F_05 | 49.050729 | 13.362756 | 2 | 1 | 1 | 3 |
| 49 | F_05_WB | Raccoon | 4.65 | Spring | F_05 | 49.050944 | 13.361986 | 2 | 3 | 2 | 6 |
| 50 | F_05_WS | Wild_Boar | 76.0 | Spring | F_05 | 49.052921 | 13.358756 | 1 | 2 | 2 | 2 |
| 51 | S_01_BB | European_Beaver | 15.5 | Summer | S_01 | 48.942082 | 13.404766 | 0 | 0 | 3 | 4 |
| 52 | S_01_DS | Badger | 9.85 | Summer | S_01 | 48.94743 | 13.40448 | 0 | 0 | 1 | 18 |
| 53 | S_01_FX | Red_Fox | 6.4 | Summer | S_01 | 48.9475 | 13.406724 | 0 | 0 | 2 | 6 |
| 54 | S_01_HR | Stoat | 0.217 | Summer | S_01 | 48.941424 | 13.405886 | 1 | 2 | 3 | 15 |
| 55 | S_01_MD | Marten | 1.45 | Summer | S_01 | 48.94788 | 13.405239 | 0 | 0 | 3 | 31 |
| 56 | S_01_RE | Roe_Deer | 19.7 | Summer | S_01 | 48.94647 | 13.405118 | 1 | 1 | 4 | 23 |
| 57 | S_01_RH | Red_Deer | 78.8 | Summer | S_01 | 48.949094 | 13.405349 | 5 | 144 | 3 | 159 |
| 58 | S_01_RT | Rat | 0.21 | Summer | S_01 | 48.940741 | 13.406834 | 0 | 0 | 2 | 9 |
| 59 | S_01_WB | Raccoon | 5.55 | Summer | S_01 | 48.94898 | 13.406162 | 0 | 0 | 1 | 8 |
| 60 | S_01_WS | Wild_Boar | 48.0 | Summer | S_01 | 48.946653 | 13.404246 | 1 | 2 | 1 | 53 |
| 61 | S_02_BB | European_Beaver | 26.6 | Summer | S_02 | 48.913056 | 13.444663 | 2 | 58 | 3 | 6 |
| 62 | S_02_DS | Badger | 8.6 | Summer | S_02 | 48.914825 | 13.444588 | 1 | 1 | 1 | 1 |
| 63 | S_02_FX | Red_Fox | 7.0 | Summer | S_02 | 48.915613 | 13.440305 | 4 | 73 | 7 | 12 |
| 64 | S_02_HR | Stoat | 0.213 | Summer | S_02 | 48.915991 | 13.446899 | 1 | 2 | 1 | 1 |
| 65 | S_02_MD | Marten | 1.25 | Summer | S_02 | 48.913035 | 13.445148 | 2 | 17 | 3 | 4 |
| 66 | S_02_RE | Roe_Deer | 28.7 | Summer | S_02 | 48.913878 | 13.439941 | 2 | 4 | 3 | 8 |
| 67 | S_02_RH | Red_Deer | 95.0 | Summer | S_02 | 48.912539 | 13.442165 | 4 | 59 | 3 | 20 |
| 68 | S_02_RT | Rat | 0.205 | Summer | S_02 | 48.914704 | 13.443262 | 0 | 0 | 2 | 2 |
| 69 | S_02_WB | Raccoon | 5.7 | Summer | S_02 | 48.917739 | 13.439883 | 1 | 5 | 1 | 2 |
| 70 | S_02_WS | Wild_Boar | 50.0 | Summer | S_02 | 48.916721 | 13.442583 | 1 | 13 | 1 | 10 |
| 71 | S_03_BB | European_Beaver | 8.5 | Summer | S_03 | 48.961496 | 13.559088 | 0 | 0 | 3 | 22 |
| 72 | S_03_DS | Badger | 8.3 | Summer | S_03 | 48.961959 | 13.553851 | 0 | 0 | 0 | 0 |
| 73 | S_03_FX | Red_Fox | 7.25 | Summer | S_03 | 48.963301 | 13.554559 | 0 | 0 | 2 | 12 |
| 74 | S_03_HR | Stoat | 0.051 | Summer | S_03 | 48.963191 | 13.555797 | 0 | 0 | 0 | 0 |
| 75 | S_03_MD | Marten | 1.15 | Summer | S_03 | 48.962201 | 13.560527 | 0 | 0 | 1 | 1 |
| 76 | S_03_RE | Roe_Deer | 20.9 | Summer | S_03 | 48.961536 | 13.557368 | 1 | 0 | 0 | 0 |
| 77 | S_03_RH | Red_Deer | 54.4 | Summer | S_03 | 48.96257 | 13.442165 | 0 | 0 | 1 | 2 |
| 78 | S_03_RT | Rat | 0.207 | Summer | S_03 | 48.964144 | 13.561538 | 1 | 0 | 1 | 3 |
| 79 | S_03_WB | Raccoon | 5.25 | Summer | S_03 | 48.962929 | 13.557313 | 0 | 0 | 2 | 2 |
| 80 | S_03_WS | Wild_Boar | 50.5 | Summer | S_03 | 48.963381 | 13.560256 | 0 | 0 | 3 | 3 |
| 81 | S_04_BB | European_Beaver | 8.65 | Summer | S_04 | 49.101218 | 13.297595 | 0 | 0 | 4 | 15 |
| 82 | S_04_DS | Badger | 6.9 | Summer | S_04 | 49.10308 | 13.2991 | 0 | 0 | 0 | 0 |
| 83 | S_04_FX | Red_Fox | 4.25 | Summer | S_04 | 49.100522 | 13.298334 | 0 | 0 | 3 | 15 |
| 84 | S_04_HR | Stoat | 0.053 | Summer | S_04 | 49.104047 | 13.295382 | 0 | 0 | 0 | 0 |
| 85 | S_04_MD | Marten | 1.2 | Summer | S_04 | 49.101522 | 13.296623 | 1 | 1 | 3 | 12 |
| 86 | S_04_RE | Roe_Deer | 11.65 | Summer | S_04 | 49.103804 | 13.296547 | 0 | 0 | 3 | 21 |
| 87 | S_04_RH | Red_Deer | 61.3 | Summer | S_04 | 49.104001 | 13.296218 | 0 | 0 | 4 | 41 |
| 88 | S_04_RT | Rat | 0.198 | Summer | S_04 | 49.10085 | 13.299053 | 0 | 0 | 0 | 0 |
| 89 | S_04_WB | Raccoon | 6.1 | Summer | S_04 | 49.103397 | 13.298238 | 0 | 0 | 1 | 4 |
| 90 | S_04_WS | Wild_Boar | 46.0 | Summer | S_04 | 49.101738 | 13.295418 | 0 | 0 | 3 | 22 |
| 91 | S_05_BB | European_Beaver | 19.3 | Summer | S_05 | 49.051653 | 13.366608 | 1 | 4 | 1 | 1 |
| 92 | S_05_DS | Badger | 7.2 | Summer | S_05 | 49.050845 | 13.365201 | 0 | 0 | 1 | 1 |
| 93 | S_05_FX | Red_Fox | 6.15 | Summer | S_05 | 49.052039 | 13.359878 | 1 | 0 | 1 | 2 |
| 94 | S_05_HR | Stoat | 0.044 | Summer | S_05 | 49.051576 | 13.36083 | 0 | 0 | 0 | 0 |
| 95 | S_05_MD | Marten | 1.8 | Summer | S_05 | 49.053314 | 13.355923 | 0 | 0 | 3 | 8 |
| 96 | S_05_RE | Roe_Deer | 28.4 | Summer | S_05 | 49.05303 | 13.356966 | 1 | 1 | 3 | 10 |
| 97 | S_05_RH | Red_Deer | 74.0 | Summer | S_05 | 49.052324 | 13.368634 | 1 | 24 | 2 | 2 |
| 98 | S_05_RT | Rat | 0.212 | Summer | S_05 | 49.050729 | 13.362756 | 2 | 1 | 5 | 32 |
| 99 | S_05_WB | Raccoon | 4.8 | Summer | S_05 | 49.050944 | 13.361986 | 0 | 0 | 2 | 3 |
| 100 | S_05_WS | Wild_Boar | 9.5 | Summer | S_05 | 49.052921 | 13.358756 | 0 | 0 | 2 | 4 |

|  | Carcass-ID | *Coleo-ptera* species num-ber | *Coleo-*  *ptera*  abun-  dance | *Diptera* species num-  ber | *Diptera* reads | *Diptera* volume [ml] | *Bacteria* species number | *Bacteria* reads | *Fungi* species num-ber | *Fungi* reads | Multi-diversity | Expo-sition  date |
| --- | --- | --- | --- | --- | --- | --- | --- | --- | --- | --- | --- | --- |
| 1 | F_01_BB | 10 | 53 | 20 | 42203 | 4.3 | 277 | 119139 | 309 | 42771 | 0.550778835 | 22 April 2021 |
| 2 | F_01_DS | 7 | 54 | 22 | 50034 | 3.8 | 268 | 224862 | 428 | 58093 | 0.396117211 | 22 April 2021 |
| 3 | F_01_FX | 15 | 195 | 24 | 16168 | 19.3 | 355 | 155108 | 306 | 118129 | 0.475911334 | 22 April 2021 |
| 4 | F_01_HR | 9 | 162 | NA | NA | 30.6 | 46 | 68492 | 232 | 12158 | 0.349976993 | 22 April 2021 |
| 5 | F_01_MD | 9 | 255 | 21 | 29465 | 0.3 | 199 | 272239 | 257 | 84088 | 0.423663875 | 22 April 2021 |
| 6 | F_01_RE | 8 | 126 | 15 | 35410 | 70.6 | 247 | 78330 | 385 | 33647 | 0.371098422 | 22 April 2021 |
| 7 | F_01_RH | 14 | 246 | 17 | 54735 | 5.6 | 164 | 97479 | 254 | 38500 | 0.452084899 | 22 April 2021 |
| 8 | F_01_RT | 4 | 14 | NA | NA | 0.3 | 84 | 84828 | 265 | 41471 | 0.182557545 | 22 April 2021 |
| 9 | F_01_WB | 8 | 177 | 35 | 45046 | 0.3 | 220 | 142971 | 321 | 112615 | 0.465210983 | 22 April 2021 |
| 10 | F_01_WS | 11 | 251 | 16 | 28128 | 151.6 | 237 | 116292 | 76 | 68634 | 0.285482983 | 22 April 2021 |
| 11 | F_02_BB | 16 | 334 | 24 | 27989 | 18.0 | 249 | 111239 | 227 | 63604 | 0.532694634 | 22 April 2021 |
| 12 | F_02_DS | 9 | 294 | 27 | 47183 | 20.3 | 328 | 240344 | 351 | 89213 | 0.514504458 | 22 April 2021 |
| 13 | F_02_FX | 18 | 281 | 8 | 62413 | 75.3 | 257 | 172660 | 532 | 189945 | 0.551384264 | 22 April 2021 |
| 14 | F_02_HR | 4 | 88 | NA | NA | 96.0 | 112 | 96530 | 255 | 40109 | 0.19918731 | 22 April 2021 |
| 15 | F_02_MD | 13 | 487 | 18 | 47745 | 15.3 | 258 | 237060 | 311 | 72171 | 0.460484035 | 22 April 2021 |
| 16 | F_02_RE | 14 | 343 | NA | NA | 123.3 | 326 | 132798 | 553 | 117052 | 0.490912433 | 22 April 2021 |
| 17 | F_02_RH | 9 | 198 | 19 | 22610 | 200.6 | 295 | 194643 | 471 | 105528 | 0.44809423 | 22 April 2021 |
| 18 | F_02_RT | 5 | 17 | 0 | 0 | 0.0 | 82 | 64235 | 204 | 24340 | 0.25147835 | 22 April 2021 |
| 19 | F_02_WB | 10 | 186 | 34 | 55341 | 0.3 | 252 | 151130 | 297 | 57092 | 0.427820937 | 22 April 2021 |
| 20 | F_02_WS | 11 | 202 | 24 | 51403 | 63.6 | 322 | 160740 | 259 | 167415 | 0.367619284 | 22 April 2021 |
| 21 | F_03_BB | 6 | 23 | 5 | 23849 | 0.8 | 131 | 122476 | 367 | 112820 | 0.247671558 | 14 May 2021 |
| 22 | F_03_DS | 8 | 316 | 19 | 54252 | 39.0 | 292 | 181578 | 297 | 85219 | 0.321195559 | 14 May 2021 |
| 23 | F_03_FX | 11 | 393 | 11 | 16813 | 88.8 | 331 | 125062 | 422 | 82865 | 0.389761542 | 14 May 2021 |
| 24 | F_03_HR | 10 | 115 | NA | NA | 8.5 | 124 | 101040 | 240 | 64736 | 0.293102027 | 14 May 2021 |
| 25 | F_03_MD | 12 | 171 | 9 | 26647 | 0.3 | 244 | 144720 | 178 | 146410 | 0.355428033 | 14 May 2021 |
| 26 | F_03_RE | 10 | 491 | 17 | 16942 | 1.1 | 391 | 164194 | 537 | 185416 | 0.486219388 | 14 May 2021 |
| 27 | F_03_RH | 16 | 161 | 4 | 16268 | 0.3 | 376 | 132652 | 323 | 137240 | 0.443444243 | 14 May 2021 |
| 28 | F_03_RT | 10 | 116 | NA | NA | 1.3 | 214 | 104572 | 99 | 35954 | 0.192444601 | 14 May 2021 |
| 29 | F_03_WB | 8 | 194 | 17 | 17393 | 0.3 | 348 | 176912 | 123 | 54116 | 0.335800909 | 14 May 2021 |
| 30 | F_03_WS | 8 | 412 | 11 | 11716 | 0.6 | 352 | 137812 | 444 | 102452 | 0.432114447 | 14 May 2021 |
| 31 | F_04_BB | 4 | 110 | 0 | 0 | 0.0 | 167 | 125355 | 220 | 69985 | 0.223444354 | 14 May 2021 |
| 32 | F_04_DS | 5 | 157 | 10 | 1245 | 0.3 | 313 | 174428 | 296 | 125178 | 0.348778368 | 14 May 2021 |
| 33 | F_04_FX | 9 | 158 | NA | NA | 0.3 | 252 | 138437 | 329 | 85090 | 0.327490169 | 14 May 2021 |
| 34 | F_04_HR | 10 | 166 | NA | NA | 0.3 | 130 | 211319 | 177 | 160158 | 0.183587793 | 14 May 2021 |
| 35 | F_04_MD | 2 | 12 | 0 | 0 | 0.0 | 110 | 98149 | 116 | 94508 | 0.086747783 | 14 May 2021 |
| 36 | F_04_RE | 6 | 131 | 0 | 0 | 0.0 | 243 | 227283 | 501 | 141332 | 0.37457935 | 14 May 2021 |
| 37 | F_04_RH | 7 | 58 | NA | NA | 0.8 | 320 | 209132 | 187 | 120734 | 0.295669707 | 14 May 2021 |
| 38 | F_04_RT | 7 | 49 | 7 | 30155 | 0.0 | 155 | 204945 | 124 | 48470 | 0.176075757 | 14 May 2021 |
| 39 | F_04_WB | 8 | 39 | 0 | 0 | 0.0 | 132 | 65329 | 158 | 53845 | 0.160829773 | 14 May 2021 |
| 40 | F_04_WS | 7 | 291 | 12 | 22992 | 0.0 | 315 | 168276 | 162 | 239378 | 0.373782474 | 14 May 2021 |
| 41 | F_05_BB | 10 | 94 | NA | NA | 8.3 | 217 | 107191 | 298 | 136219 | 0.245145577 | 14 May 2021 |
| 42 | F_05_DS | 10 | 158 | 26 | 20344 | 0.3 | 217 | 133890 | 176 | 65323 | 0.355295664 | 14 May 2021 |
| 43 | F_05_FX | 11 | 230 | 8 | 11441 | 8.0 | 346 | 132931 | 349 | 155926 | 0.365138287 | 14 May 2021 |
| 44 | F_05_HR | 6 | 76 | NA | NA | 0.3 | 91 | 177399 | 183 | 57335 | 0.206023447 | 14 May 2021 |
| 45 | F_05_MD | 10 | 267 | NA | NA | 0.3 | 205 | 148165 | 248 | 71327 | 0.299435874 | 14 May 2021 |
| 46 | F_05_RE | 13 | 172 | NA | NA | 5.3 | 236 | 151181 | 343 | 69302 | 0.324522438 | 14 May 2021 |
| 47 | F_05_RH | 8 | 149 | NA | NA | 0.3 | 209 | 127070 | 452 | 127517 | 0.344786668 | 14 May 2021 |
| 48 | F_05_RT | 11 | 66 | 0 | 0 | 0.0 | 241 | 239022 | 334 | 128499 | 0.362542829 | 14 May 2021 |
| 49 | F_05_WB | 5 | 47 | 0 | 0 | 0.0 | 232 | 237433 | 507 | 90658 | 0.372590029 | 14 May 2021 |
| 50 | F_05_WS | 11 | 218 | 16 | 19241 | 1.0 | 211 | 64280 | 335 | 43307 | 0.400854629 | 14 May 2021 |
| 51 | S_01_BB | 14 | 42 | 33 | 44644 | 0.0 | 104 | 43714 | 333 | 56026 | 0.443108053 | 06 July 2021 |
| 52 | S_01_DS | 6 | 16 | 29 | 55032 | 3.3 | 366 | 101832 | 549 | 158419 | 0.453780034 | 06 July 2021 |
| 53 | S_01_FX | 9 | 279 | 22 | 41751 | 5.6 | 142 | 54795 | 130 | 58588 | 0.293614277 | 06 July 2021 |
| 54 | S_01_HR | 7 | 66 | 7 | 22772 | 0.9 | 310 | 84558 | 486 | 124236 | 0.42852237 | 06 July 2021 |
| 55 | S_01_MD | 7 | 79 | 10 | 31852 | 0.3 | 149 | 60168 | 343 | 31374 | 0.313148474 | 06 July 2021 |
| 56 | S_01_RE | 7 | 72 | 23 | 44233 | 6.0 | 381 | 66273 | 583 | 151011 | 0.560086157 | 06 July 2021 |
| 57 | S_01_RH | 10 | 75 | 30 | 73161 | 40.3 | 368 | 91993 | 508 | 227115 | 0.698775153 | 06 July 2021 |
| 58 | S_01_RT | 7 | 96 | 0 | 0 | 0.0 | 147 | 44610 | 312 | 73004 | 0.244366298 | 06 July 2021 |
| 59 | S_01_WB | 12 | 194 | 29 | 41469 | 0.5 | 434 | 101391 | 476 | 50008 | 0.513946419 | 06 July 2021 |
| 60 | S_01_WS | 16 | 143 | 35 | 65395 | 0.3 | 282 | 153509 | 362 | 294535 | 0.523792852 | 06 July 2021 |
| 61 | S_02_BB | 12 | 143 | 33 | 40244 | 12.0 | 185 | 98517 | 499 | 185309 | 0.562417225 | 06 July 2021 |
| 62 | S_02_DS | 13 | 107 | 31 | 27971 | 25.0 | 347 | 69885 | 642 | 132421 | 0.576734355 | 06 July 2021 |
| 63 | S_02_FX | 13 | 216 | 42 | 67067 | 50.6 | 449 | 134233 | 617 | 95165 | 0.888299471 | 06 July 2021 |
| 64 | S_02_HR | 14 | 499 | 6 | 11181 | 130.6 | 119 | 69413 | 322 | 94326 | 0.333338097 | 06 July 2021 |
| 65 | S_02_MD | 7 | 171 | 25 | 28337 | 1.0 | 269 | 93608 | 472 | 84849 | 0.509232105 | 06 July 2021 |
| 66 | S_02_RE | 17 | 279 | 23 | 18125 | 70.9 | 351 | 142181 | 330 | 48999 | 0.586127382 | 06 July 2021 |
| 67 | S_02_RH | 14 | 212 | 25 | 67816 | 34.6 | 298 | 156392 | 527 | 50384 | 0.665040393 | 06 July 2021 |
| 68 | S_02_RT | 12 | 41 | 0 | 0 | 0.0 | 352 | 96430 | 503 | 103025 | 0.411279797 | 06 July 2021 |
| 69 | S_02_WB | 14 | 362 | 12 | 47849 | 0.3 | 417 | 120317 | 573 | 91751 | 0.523495286 | 06 July 2021 |
| 70 | S_02_WS | 12 | 94 | 46 | 45356 | 5.0 | 335 | 129463 | 398 | 57247 | 0.5543212 | 06 July 2021 |
| 71 | S_03_BB | 3 | 7 | 31 | 54707 | 0.0 | 481 | 255881 | 284 | 105771 | 0.45191979 | 07 July 2021 |
| 72 | S_03_DS | 6 | 99 | 33 | 47942 | 0.0 | 272 | 135176 | 278 | 105500 | 0.341539168 | 07 July 2021 |
| 73 | S_03_FX | 10 | 115 | 16 | 98863 | 0.0 | 155 | 106274 | 313 | 69114 | 0.333146699 | 07 July 2021 |
| 74 | S_03_HR | 2 | 9 | NA | NA | 0.3 | 81 | 114240 | 229 | 35099 | 0.106034683 | 07 July 2021 |
| 75 | S_03_MD | 7 | 28 | 29 | 42798 | 0.0 | 372 | 125095 | 277 | 91218 | 0.394505627 | 07 July 2021 |
| 76 | S_03_RE | 4 | 14 | 21 | 15422 | 1.0 | 414 | 200412 | 262 | 68420 | 0.357925085 | 07 July 2021 |
| 77 | S_03_RH | 5 | 32 | 30 | 88920 | 0.0 | 347 | 122820 | 252 | 84063 | 0.364457653 | 07 July 2021 |
| 78 | S_03_RT | 5 | 6 | 11 | 24066 | 0.0 | 225 | 170506 | 151 | 122007 | 0.260457219 | 07 July 2021 |
| 79 | S_03_WB | 9 | 53 | 24 | 20980 | 0.3 | 314 | 121335 | 218 | 63110 | 0.383303989 | 07 July 2021 |
| 80 | S_03_WS | 5 | 15 | 25 | 44511 | 0.6 | 427 | 248986 | 236 | 143034 | 0.4175271 | 07 July 2021 |
| 81 | S_04_BB | 12 | 87 | 24 | 70996 | 160.0 | 138 | 55752 | 213 | 82445 | 0.396418726 | 07 July 2021 |
| 82 | S_04_DS | 9 | 20 | 42 | 26201 | 140.0 | 254 | 114141 | 79 | 44173 | 0.344027161 | 07 July 2021 |
| 83 | S_04_FX | 17 | 202 | 12 | 45758 | 0.8 | 343 | 181059 | 300 | 66257 | 0.469045478 | 07 July 2021 |
| 84 | S_04_HR | 13 | 278 | 7 | 19818 | 7.5 | 37 | 36655 | 59 | 13245 | 0.173869921 | 07 July 2021 |
| 85 | S_04_MD | 2 | 73 | 6 | 549 | 0.6 | 208 | 91658 | 382 | 164446 | 0.316260889 | 07 July 2021 |
| 86 | S_04_RE | 11 | 242 | 11 | 65644 | 4.3 | 292 | 164343 | 251 | 57959 | 0.379474552 | 07 July 2021 |
| 87 | S_04_RH | 7 | 88 | 27 | 95643 | 70.6 | 410 | 167197 | 366 | 175674 | 0.494959715 | 07 July 2021 |
| 88 | S_04_RT | 6 | 12 | 9 | 707 | 0.0 | 192 | 119878 | 333 | 84402 | 0.241140916 | 07 July 2021 |
| 89 | S_04_WB | 6 | 20 | 24 | 17642 | 0.0 | 314 | 145520 | 139 | 33123 | 0.31120786 | 07 July 2021 |
| 90 | S_04_WS | 10 | 87 | 29 | 48129 | 2.0 | 318 | 167230 | 295 | 125456 | 0.455864331 | 07 July 2021 |
| 91 | S_05_BB | 8 | 28 | 31 | 82490 | 50.3 | 360 | 129310 | 384 | 95924 | 0.46796437 | 07 July 2021 |
| 92 | S_05_DS | 11 | 59 | 32 | 102236 | 10.0 | 244 | 45261 | 310 | 101305 | 0.406627163 | 07 July 2021 |
| 93 | S_05_FX | 12 | 175 | 11 | 31112 | 505.0 | 238 | 66620 | 337 | 101670 | 0.378063143 | 07 July 2021 |
| 94 | S_05_HR | 9 | 149 | NA | NA | 90.3 | 118 | 82480 | 173 | 41530 | 0.169132108 | 07 July 2021 |
| 95 | S_05_MD | 10 | 65 | 11 | 11737 | 0.6 | 176 | 50258 | 398 | 76768 | 0.368183247 | 07 July 2021 |
| 96 | S_05_RE | 16 | 103 | 6 | 19748 | 647.5 | 353 | 110682 | 392 | 145543 | 0.498729122 | 07 July 2021 |
| 97 | S_05_RH | 11 | 112 | 24 | 47840 | 440.3 | 283 | 34205 | 326 | 114372 | 0.452451713 | 07 July 2021 |
| 98 | S_05_RT | 11 | 132 | 10 | 1941 | 0.6 | 267 | 60765 | 223 | 65052 | 0.474205618 | 07 July 2021 |
| 99 | S_05_WB | 9 | 190 | 14 | 29654 | 0.5 | 229 | 75494 | 259 | 186197 | 0.328263397 | 07 July 2021 |
| 100 | S_05_WS | 14 | 101 | 26 | 69316 | 140.6 | 240 | 58863 | 540 | 167502 | 0.494798575 | 07 July 2021 |

**Table S2.** Beta regression model showing the relationship between variables studied (“Season” and “Carcass mass”) and response variable (“Multidiversity”). An increase in multidiversity with carcass mass was statistically significant in spring and summer. The slope of multidiversity values was significantly steeper in summer than in spring. The estimate of the parameters, the standard error of the parameters (SE), the z-value, and the p-value (Pr(>|z|)) are shown. Significant p-values (< 0.05) are indicated in boldface.

| Response variable | Model | Parameter | Estimate ± SE | z-value | Pr(>\|z\|) |
| --- | --- | --- | --- | --- | --- |
| Multidiversity | Season:log(Carcass mass) | Intercept | -0.70 ± 0.06 | -11.45 | **< 0.001** |
|  | Spring:log(Carcass mass) | | 0.08 ± 0.02 | 3.18 | **0.0014** |
|  | Summer:log(Carcass mass) | | 0.20 ± 0.02 | 7.08 | **< 0.001** |
|  | Season*log(Carcass mass) | Summer | 0.24 ± 0.12 | 2.02 | **0.0427** |

**Table S3.** Exemplary negative binomial generalized linear model (NBGLM) showing the relationship between variables studied (“Abundance” of all other taxa) and response variable (“Species number”). The estimate of the parameters, the standard error of the parameters (SE), the z-value, and the p-value (Pr(>|z|)) are shown. Significant p-values (< 0.05) are indicated in boldface.

| Response variable | Model | Parameter | Estimate ± SE | z-value | Pr(>\|z\|) |
| --- | --- | --- | --- | --- | --- |
| Species number  *Diptera* |  |  |  |  |  |
|  | log(Abundance mammals+1) + log(Abundance birds+1) +  log(Abundance *Coleoptera*) + log(Abundance *Bacteria*) +  log(Abundance *Fungi*) | Intercept | 3.03 ± 1.71 | 1.76 | 0.0771 |
|  | log(Abundance mammals+1) | | 0.04 ± 0.04 | 0.91 | 0.3614 |
|  | log(Abundance birds + 1) | | 0.08 ± 0.04 | 1.78 | 0.0743 |
|  | log(Abundance *Coleoptera*) | | -0.14 ± 0.05 | -2.51 | **0.0119** |
|  | log(Abundance *Bacteria*) | | 0.05 ± 0.12 | 0.45 | 0.6493 |
|  | log(Abundance *Fungi*) | | -0.01 ± 0.10 | -0.14 | 0.8886 |

##

## Appendix S2 Comprehensive description of biodiversity sampling

### Vertebrates

To monitor carrion-visiting vertebrates, one camera trap (RECONYX Ultra Fire XR6) was installed at each carcass site and positioned 5 – 6 m from the carcass with a clear field of view (Stiegler et al. 2020). It was attached to a tree at 60 – 80 cm height above the ground. One hind limb of each carcass was tied to a wooden pole with a cord to prevent the carcass from being dragged out of the camera’s field of view (von Hoermann et al. 2021). Each camera trap was operating for an observation period of 30 days. If animals moved the carcass out of the camera’s field of view the affected photos were discarded (Turner et al. 2017, Stiegler et al. 2020). All remaining images were processed with the software TRAPPER (Bubnicki et al. 2016), which allowed a manual classification of the recorded carcass-visiting species by experts. Carcass visitors were defined as all animals approaching carrion (e.g., scavengers, predators lured by the carcass, or random appearances; Stiegler et al. 2020). To avoid temporal autocorrelation, the raw species observations were converted to independent “events”, which were used for further analyses (O’Brien et al. 2003, Li et al. 2010, Stiegler et al. 2020). An event was defined as a consecutive sequence of images of the same species at the same camera trap location, with a minimum interval of 30 minutes from the previous and subsequent image sequence showing the same species at the same location.

### Insects

Carrion-associated insects were sampled with one pitfall trap installed adjacent to the mouth at each carcass to cover an important settlement area for carrion-inhabiting insects (Dekeirsschieter et al. 2011, von Hoermann et al. 2021, 2023). The pitfall traps were composed of two transparent ground-level plastic cups stacked inside each other (half-liter PLA cups, diameter of 95 mm, height of 151.2 mm; Huhtamaki Foodservice GmbH, Alf/Mosel, Germany; von Hoermann et al. 2021). The inner cup was filled with a soapy solution (water and a drop of odorless detergent; Klar EcoSensitive, AlmaWin, Winterbach, Germany) for surface tension reduction. The cups were perforated to prevent overflow by rain and the opening of the trap was covered with a moss cushion. During the decomposition period, a total of four collection events per carcass and trap were conducted on days 4, 8, 16, and 23 after exposure to cover all decomposition stages (fresh, bloated, post-bloating, advanced decay, and dry remains; based on proved succession data; Matuszewski et al. 2010, 2011, Benbow et al. 2015, von Hoermann et al. 2018, 2020). The pitfall traps were opened 48 h before content collection to guarantee a consistent sample period for each trapping event (von Hoermann et al. 2023). During each event, photographs of the carcass were taken for later morphological evaluation and classification of decomposition stages (von Hoermann et al. 2021, 2023). Immediately after emptying the pitfall trap, all collected invertebrates were preserved in 70% ethanol (VWR International GmbH, Darmstadt, Germany) and then presorted into larger taxonomic groups (family-level based). After presorting, beetle individuals from the taxa Dermestidae, Histeridae, Scarabaeidae, Silphidae, Staphylinidae, and “Coleoptera rest” (all other beetle taxonomic groups) were identified to the species level by external taxonomic experts from bureaus (Private Entomological Laboratory & Collection in Prague-West, Ostrava Museum in Ostrava, Bavarian State Collection for Zoology in Munich, Czech University of Life Sciences in Prague, and Charles University in Prague) and stored at the BFNP. The sampling campaign resulted in 400 sample units (four collection events per carcass for ten carcasses x five sites per season), which formed the basis for subsequent statistical analyses.

The total volume of the Diptera larvae contents of each sample retrieved from the pitfall traps was measured (152 sample units). Diptera species were identified by DNA metabarcoding (conducted by AIM Advanced Identification Methods GmbH), where Amplicon Sequencing Variants (ASVs, represented by Barcode Index Numbers (BINs) that closely approximate species; Ratnasingham & Hebert 2013) were generated. Based on the genus and family level, carrion-related dipterans were selected for subsequent analyses. The number of reads per BIN was used as a substitute for abundance.

### Microbes

The carrion-inhabiting microbiome was sampled with swabs of the carcass oral mucosa on days 0, 4, 8, 16, and 23 after exposure (von Hoermann et al. 2022, 2023). A defined swab protocol (three “in and out”-movements) was performed with a sterile cotton applicator (Copan FLOQSwabsTM, MAST Diagnostica GmbH, Reinfeld, Germany) in the upper palate of the mouth and under the tongue, respectively (von Hoermann et al. 2022, 2023). After sample collection, each cotton swab with the adherent microbiome DNA material was stored in 600 μl RNA*later*® medium with immediate RNase inactivation (RNA*later*®, Merck, Darmstadt, Germany; von Hoermann et al. 2022, 2023). The sampling resulted in 448 bacterial sample units and 430 fungal sample units with sufficient DNA for further processing. The bacterial and fungal diversity in the DNA samples was characterized with amplicon sequencing (following the procedure described in von Hoermann et al. 2023). Total genomic DNA was isolated using a PowerMag Soil DNA extraction kit (Qiagen, with an addition of 15 mg/ml lysozyme during lysis). To characterize the microbial species, two gene regions that have previously been successful in terrestrial vertebrate carrion studies were selected for amplicon sequencing (Metcalf et al. 2016). The bacterial community composition was determined by PCR amplification (using the primers 515f and 806r with a dual-indexing strategy; Caporaso et al. 2012, Kozich et al. 2013) and high-throughput sequencing of the 16S rRNA gene (V4 region; Hird 2017, Tláskal et al. 2017, Müller et al. 2020, von Hoermann et al. 2023). Fungi were sampled from the same carrion oral mucosa swabs following the procedure described for bacteria. High-throughput sequencing was based on the amplification of the fungal internal transcribed spacer (ITS) region (Lindahl et al. 2013, Hagge et al. 2019, Müller et al. 2020). Amplification of the ITS region was conducted with the fungal primers gITS7 and ITS4 with added Fluidigm indexing oligonucleotides (CS1/CS2; Manter & Vivanco 2007, Ihrmark et al. 2012) in a primary PCR reaction, followed by a cleanup with a QIAQuick PCR Purification kit (Qiagen). The resulting products were normalized and pooled by the MSU RTSF Genomics Core (Michigan State University, USA) and afterward sequenced on an Illumina MiSeq platform (Illumina, San Diego, CA, USA, 2 x 250 bp reads). Initial filtering and demultiplexing were performed for bacterial sequence reads by using DADA2 implemented in QIIME 2 (Bokulich et al. 2013, Callahan et al. 2016, Bolyen et al. 2019). Bacterial Operational Taxonomic Units (OTUs, as a close approximation to species) were taxonomically assigned with a naïve Bayes classifier and the SILVA reference database (v 13.2, 99%; Quast et al. 2013, Bokulich et al. 2018). Fungal sequence reads were subjected to similar processing and demultiplexing protocols within QIIME 2 as bacteria, except the UNITE reference (alpha version 8.2 UNITE 99% similarity reference set) was used to taxonomically assign fungal OTUs (Bates et al. 2013, Nilsson et al. 2019, Abarenkov et al. 2020). Sequencing data for this project was deposited in the NCBI Sequence Read Archive (SRA). In subsequent analyses, the number of reads per OTU was used as a substitute for abundance, as is done in routine postmortem microbiome studies (Pechal et al. 2018).

**References**

Abarenkov, Kessy, Allan Zirk, Timo Piirmann, Raivo Pöhönen, Filipp Ivanov, R. Henrik Nilsson, and Urmas Kõljalg. 2020. “UNITE QIIME Release for Fungi.” Application/gzip. UNITE Community.

Bates, Scott T., Steven Ahrendt, Holly M. Bik, Thomas D. Bruns, J. Gregory Caporaso, James Cole, Michael Dwan, et al. 2013. “Meeting Report: Fungal ITS Workshop (October 2012).” *Standards in Genomic Sciences* 8 (1): 118–23.

Benbow, M. Eric, Jeffery K. Tomberlin, and Aaron M. Tarone, eds. 2015. *Carrion Ecology, Evolution, and Their Applications*. Boca Raton: CRC Press.

Bokulich, Nicholas A., Benjamin D. Kaehler, Jai Ram Rideout, Matthew Dillon, Evan Bolyen, Rob Knight, Gavin A. Huttley, and J. Gregory Caporaso. 2018. “Optimizing Taxonomic Classification of Marker-Gene Amplicon Sequences with QIIME 2’s Q2-Feature-Classifier Plugin.” *Microbiome* 6 (1): 90.

Bokulich, Nicholas A., Sathish Subramanian, Jeremiah J. Faith, Dirk Gevers, Jeffrey I. Gordon, Rob Knight, David A. Mills, and J. Gregory Caporaso. 2013. “Quality-Filtering Vastly Improves Diversity Estimates from Illumina Amplicon Sequencing.” *Nature Methods* 10 (1): 57–59.

Bolyen, Evan, Jai Ram Rideout, Matthew R. Dillon, Nicholas A. Bokulich, Christian C. Abnet, Gabriel A. Al-Ghalith, Harriet Alexander, et al. 2019. “Reproducible, Interactive, Scalable and Extensible Microbiome Data Science Using QIIME 2.” *Nature Biotechnology* 37 (8): 852–57.

Bubnicki, Jakub W., Marcin Churski, and Dries P. J. Kuijper. 2016. “Trapper: An Open Source Web-Based Application to Manage Camera Trapping Projects.” *Methods in Ecology and Evolution* 7 (10): 1209–16.

Callahan, Benjamin J., Paul J. McMurdie, Michael J. Rosen, Andrew W. Han, Amy Jo A. Johnson, and Susan P. Holmes. 2016. “DADA2: High-Resolution Sample Inference from Illumina Amplicon Data.” *Nature Methods* 13 (7): 581–83.

Caporaso, J. Gregory, Christian L. Lauber, William A. Walters, Donna Berg-Lyons, James Huntley, Noah Fierer, Sarah M. Owens, et al. 2012. “Ultra-High-Throughput Microbial Community Analysis on the Illumina HiSeq and MiSeq Platforms.” *The ISME Journal* 6 (8): 1621–24.

Dekeirsschieter, Jessica, François J. Verheggen, Eric Haubruge, and Yves Brostaux. 2011. “Carrion Beetles Visiting Pig Carcasses during Early Spring in Urban, Forest and Agricultural Biotopes of Western Europe.” *Journal of Insect Science* 11 (1): 73.

Hagge, Jonas, Claus Bässler, Axel Gruppe, Björn Hoppe, Harald Kellner, Franz-Sebastian Krah, Jörg Müller, Sebastian Seibold, Elisa Stengel, and Simon Thorn. 2019. “Bark Coverage Shifts Assembly Processes of Microbial Decomposer Communities in Dead Wood.” *Proceedings. Biological Sciences* 286 (1912): 20191744.

Hird, Sarah M. 2017. “Evolutionary Biology Needs Wild Microbiomes.” *Frontiers in Microbiology* 8.

Hoermann, Christian von, Dennis Jauch, Carolin Kubotsch, Kirsten Reichel-Jung, Sandra Steiger, and Manfred Ayasse. 2018. “Effects of Abiotic Environmental Factors and Land Use on the Diversity of Carrion-Visiting Silphid Beetles (Coleoptera: Silphidae): A Large Scale Carrion Study.” *PloS One* 13 (5): e0196839.

Hoermann, Christian von, Sandra Weithmann, Markus Deißler, Manfred Ayasse, and Sandra Steiger. 2020. “Forest Habitat Parameters Influence Abundance and Diversity of Cadaver-Visiting Dung Beetles in Central Europe.” *Royal Society Open Science* 7 (3): 191722.

Hoermann, Christian von, Tomáš Lackner, David Sommer, Marco Heurich, M. Eric Benbow, and Jörg Müller. 2021. “Carcasses at Fixed Locations Host a Higher Diversity of Necrophilous Beetles.” *Insects* 12 (5): 412.

Hoermann, Christian von, Sandra Weithmann, Johannes Sikorski, Omer Nevo, Krzysztof Szpila, Andrzej Grzywacz, Jan-Eric Grunwald, et al. 2022. “Linking Bacteria, Volatiles and Insects on Carrion: The Role of Temporal and Spatial Factors Regulating Inter-Kingdom Communication via Volatiles.” *Royal Society Open Science* 9 (8): 220555.

Hoermann, Christian von, M. Eric Benbow, Ann-Marie Rottler-Hoermann, Tomáš Lackner, David Sommer, Joseph P. Receveur, Claus Bässler, Marco Heurich, and Jörg Müller. 2023. “Factors Influencing Carrion Communities Are Only Partially Consistent with Those of Deadwood Necromass.” *Oecologia*, January.

Ihrmark, Katarina, Inga T. M. Bödeker, Karelyn Cruz-Martinez, Hanna Friberg, Ariana Kubartova, Jessica Schenck, Ylva Strid, et al. 2012. “New Primers to Amplify the Fungal ITS2 Region--Evaluation by 454-Sequencing of Artificial and Natural Communities.” *FEMS Microbiology Ecology* 82 (3): 666–77.

Kozich, James J., Sarah L. Westcott, Nielson T. Baxter, Sarah K. Highlander, and Patrick D. Schloss. 2013. “Development of a Dual-Index Sequencing Strategy and Curation Pipeline for Analyzing Amplicon Sequence Data on the MiSeq Illumina Sequencing Platform.” *Applied and Environmental Microbiology* 79 (17): 5112–20.

Li, Sheng, William J. Mcshea, Dajun Wang, Liangkun Shao, and Xiaogang Shi. 2010. “The Use of Infrared-Triggered Cameras for Surveying Phasianids in Sichuan Province, China.” *Ibis* 152 (2): 299–309.

Lindahl, Björn D., R. Henrik Nilsson, Leho Tedersoo, Kessy Abarenkov, Tor Carlsen, Rasmus Kjøller, Urmas Kõljalg, et al. 2013. “Fungal Community Analysis by High-Throughput Sequencing of Amplified Markers – a User’s Guide.” *New Phytologist* 199 (1): 288–99.

Manter, Daniel, and Jorge Vivanco. 2007. “Use of the ITS Primers, ITS1F and ITS4, to Characterize Fungal Abundance and Diversity in Mixed-Template Samples by qPCR and Length Heterogeneity Analysis.” *Journal of Microbiological Methods* 71 (November): 7–14.

Matuszewski, Szymon, Daria Bajerlein, Szymon Konwerski, and Krzysztof Szpila. 2010. “Insect Succession and Carrion Decomposition in Selected Forests of Central Europe. Part 2: Composition and Residency Patterns of Carrion Fauna.” *Forensic Science International* 195 (1–3): 42–51.

———. 2011. “Insect Succession and Carrion Decomposition in Selected Forests of Central Europe. Part 3: Succession of Carrion Fauna.” *Forensic Science International* 207 (1): 150–63.

Metcalf, Jessica L., Zhenjiang Zech Xu, Sophie Weiss, Simon Lax, Will Van Treuren, Embriette R. Hyde, Se Jin Song, et al. 2016. “Microbial Community Assembly and Metabolic Function during Mammalian Corpse Decomposition.” *Science (New York, N.Y.)* 351 (6269): 158–62.

Müller, Jörg, Mike Ulyshen, Sebastian Seibold, Marc Cadotte, Anne Chao, Claus Bässler, Sebastian Vogel, et al. 2020. “Primary Determinants of Communities in Deadwood Vary among Taxa but Are Regionally Consistent.” *Oikos* 129 (10): 1579–88.

Nilsson, Rolf Henrik, Karl-Henrik Larsson, Andy F. S. Taylor, Johan Bengtsson-Palme, Thomas S. Jeppesen, Dmitry Schigel, Peter Kennedy, et al. 2019. “The UNITE Database for Molecular Identification of Fungi: Handling Dark Taxa and Parallel Taxonomic Classifications.” *Nucleic Acids Research* 47 (D1): D259–64.

O’Brien, Timothy G., Margaret F. Kinnaird, and Hariyo T. Wibisono. 2003. “Crouching Tigers, Hidden Prey: Sumatran Tiger and Prey Populations in a Tropical Forest Landscape.” *Animal Conservation* 6 (2): 131–39.

Pechal, Jennifer L., Carl J. Schmidt, Heather R. Jordan, and M. Eric Benbow. 2018. “A Large-Scale Survey of the Postmortem Human Microbiome, and Its Potential to Provide Insight into the Living Health Condition.” *Scientific Reports* 8 (1): 5724.

Quast, Christian, Elmar Pruesse, Pelin Yilmaz, Jan Gerken, Timmy Schweer, Pablo Yarza, Jörg Peplies, and Frank Oliver Glöckner. 2013. “The SILVA Ribosomal RNA Gene Database Project: Improved Data Processing and Web-Based Tools.” *Nucleic Acids Research* 41 (D1): D590–96.

Ratnasingham, Sujeevan, and Paul D. N. Hebert. 2013. “A DNA-Based Registry for All Animal Species: The Barcode Index Number (BIN) System.” *PloS One* 8 (7): e66213.

Stiegler, Jonas, Christian von Hoermann, Jörg Müller, M. Eric Benbow, and Marco Heurich. 2020. “Carcass Provisioning for Scavenger Conservation in a Temperate Forest Ecosystem.” *Ecosphere* 11 (4): e03063.

Tláskal, Vojtech, Petra Zrustová, Tomáš Vrška, and Petr Baldrian. 2017. “Bacteria Associated with Decomposing Dead Wood in a Natural Temperate Forest.” *FEMS Microbiology Ecology* 93 (12).

Turner, Kelsey L., Erin F. Abernethy, L. Mike Conner, Olin E. Rhodes Jr., and James C. Beasley. 2017. “Abiotic and Biotic Factors Modulate Carrion Fate and Vertebrate Scavenging Communities.” *Ecology* 98 (9): 2413–24.
